# A complementary approach to conjugated *N*-acyliminium formation through photoredox-catalyzed intermolecular radical addition to allenamides and allencarbamates

**DOI:** 10.3762/bjoc.16.165

**Published:** 2020-08-12

**Authors:** Olusesan K Koleoso, Matthew Turner, Felix Plasser, Marc C Kimber

**Affiliations:** 1 School of Science, Department of Chemistry, Loughborough University, Loughborough, LE11 3TU, UK; 2 Department of Pharmaceutical Technology, Moshood Abiola Polytechnic, Abeokuta, Nigeria

**Keywords:** allenamide, allene, intermolecular, *N*-acyliminium, photoredox

## Abstract

An intermolecular radical addition, using photoredox catalysis, to allenamides and allencarbamates is reported. This transformation synthesizes *N*-acyl-*N’*-aryl-*N*,*N’*-allylaminals, and proceeds by a conjugated *N*-acyliminium intermediate that previously has principally been generated by electrophilic activation methods. The radical adds to the central carbon of the allene giving a conjugated *N*-acyliminium that undergoes nucleophilic addition by arylamines and alcohols.

## Introduction

**Scheme 1:**
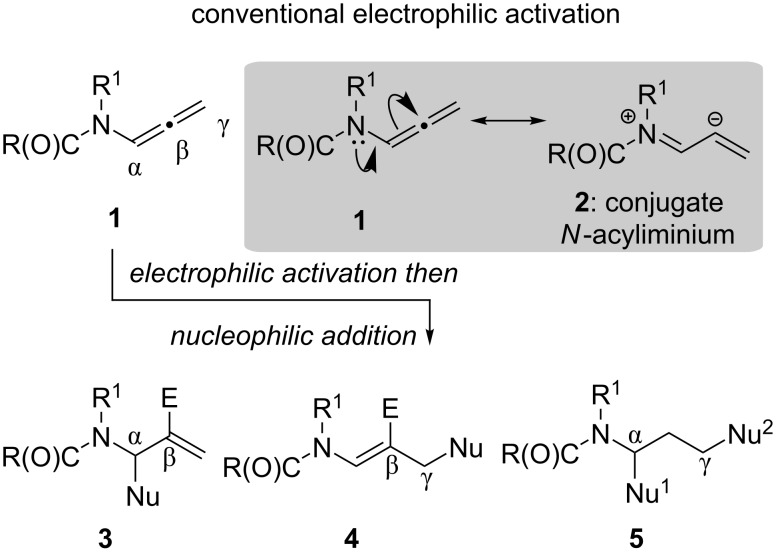
Electrophilic activation of allenamides.

Allenamides (Scheme 1, **1**) and their congeners have attracted considerable attention over the past 15 years due to their characteristic reactivity profiles [1–4]. The reactivity that an allenamide can display is distinct from a traditional allene due to the presence of an amide unit attached at the α-carbon. This substituent can donate electron density into the allene, principally onto the central β-carbon, that can be harnessed in subsequent chemical transformations leading to regiochemical confidence in the resulting products (Scheme 1).

Importantly, this unique reactivity has underpinned allenamide chemistry and led to the development of a number of innovative transformations, including cycloadditions [5–9], intramolecular cyclizations and intermolecular addition reactions [10–18], as well as the use of the allenamide building block in natural product synthesis [1].

Addition reactions of allenamides, which can also encompass intramolecular cyclizations, are typically promoted by the electrophilic activation of the β-carbon of the allenamide. This can be achieved using various electrophilic methods, including the use of Brönsted acids [15,19–22], halogenation sources [16,18,23–28], by means of oxidation [20,29–31] or through the use of a transition metal such as Au(I) [8,10–16,32–36]. The reaction of the allenamide with an electrophilic source promotes the formation of a conjugated *N*-acyliminium intermediate **2** [37–40] that subsequently undergoes an addition reaction with a suitable nucleophile (Scheme 1).

**Scheme 2:**
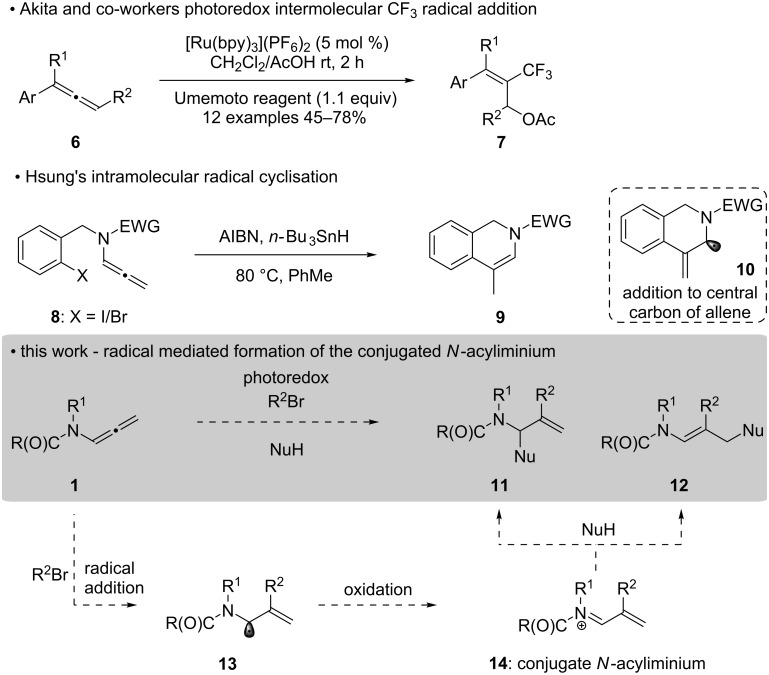
The planned intramolecular radical addition to allenamides generating the conjugated *N*-acyliminium intermediate **14**.

Importantly, the electrophilic mediated formation of this key intermediate **2** have been the cornerstone for allenamide chemistry over the past 15 years. Therefore, given the importance of the allenamide building block we sought a new synthetic methodology that could generate this conjugated *N*-acyliminium intermediate **2**, that would not be dependent on conventional electrophilic activation modes (Scheme 2).

**Scheme 3:**
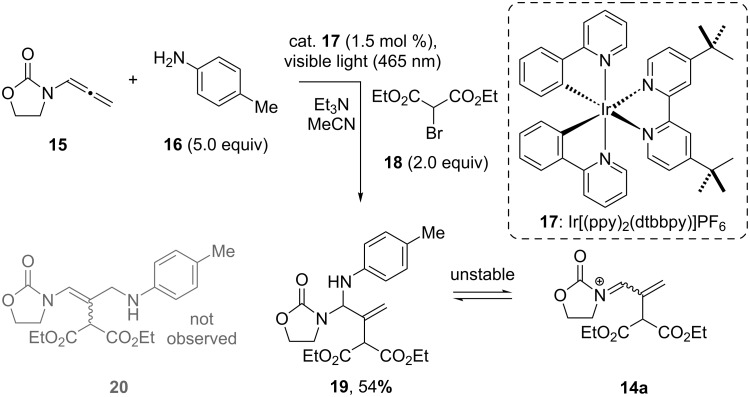
Photoredox Ir-catalyzed intermolecular addition of bromide **18** and aniline **16** to allenamide **15**.

In principle, a new method for its generation could greatly enhance the value of the allenamide building block, as well as potentially unlocking new chemical reactivity yet unseen in the context of electrophilic activation. We envisaged that a route to the conjugated *N*-acyliminium intermediate could be achieved via an intermolecular addition of an electrophilic radical to an allenamide (**1**) followed by an oxidation process on the subsequently formed radical (Scheme 3) [41]. The thought process behind this approach is based on three observations; (i) Akita and co-workers disclosure on the photoredox-catalyzed oxytrifluoromethylation of allenes **6** to give 2-trifluoromethylated allyl acetates **7** [42–44]; (ii) that intramolecular radical cyclization of allenamides have been reported by Hsung and co-workers, where the radical adds principally to the central carbon of the allene (**10**) [45]; and (iii) the photoredox-catalyzed addition of radicals to enamides reported by Masson [46–49], and more recently by our own laboratory in the synthesis of *N*,*N’*-aminals [50]. Therefore, photoredox-catalysis would be employed to generate an electrophilic radical that would add to the central carbon of the allenamide **1** to give a transient radical **13**, whose oxidation, facilitated by the photoredox catalyst [47–48], would provide the conjugated *N*-acyliminium **14**. The iminium **14** could then undergo traditional nucleophilic addition giving the addition product. Given that the iridium complex Ir[(ppy)_2_(dtbbpy)]PF_6_ had been effective [47–48,50] in this radical/cationic pathway with enamides we would now like to report our preliminary findings on the performance of allenamides. Specifically, the electron-withdrawing group on the allenamide and the nucleophile is examined. We provide evidence for the formation of the *N*-acyliminium intermediate through direct sample loop and flow injection analysis by HRESIMS, and DFT analysis of the *N*-acyliminium intermediate is provided to explain the addition product distribution.

## Results and Discussion

We began our study by using the iridium catalyst Ir[(ppy)_2_(dtbbpy)]PF_6_ (**17**) and the reaction conditions used in the addition of amine nucleophiles to enamides [48,50]. By exploiting these reaction conditions, using diethyl bromomalonate (**18**), 4-anisidine (**16**) as the amine nucleophile, and allenamide **15** we were able to isolate *N*-acyl-*N*’-aryl-*N*,*N*’-allylaminal **19** as the sole identifiable product in 54% yield.

All of the starting allenamide **15** was consumed in the reaction, but the isolation of **19** proved very challenging and we can account for this instability by the identification of **14a** in the ^1^H NMR spectrum of the crude reaction mixture. A comparable instability profile was observed in the formation of *N*-acyl-*N*’-aryl-*N*,*N*’-aminals derived from enamides [50]; however, to our knowledge, there are no reports of the isolation of analogous *N*-acyl-*N*’-aryl-*N*,*N*’-allylaminal substrates in the literature, consequently, these specific conditions were not optimized [51]. The evidence for the formation of **19** was the appearance of the methylene protons in ^1^H NMR at δ 5.46 (d, *J* = 1.6 Hz, 1H) and δ 5.45 (d, *J* = 2.0 Hz, 1H) ppm, respectively. Crucially, it is apparent that using the photoredox conditions described in Scheme 3, the aniline nucleophile adds primarily at the α-position of the allenamide **15**; this is in contrast to the archetypal electrophilic activation modes where comparable nucleophiles add to the γ-position [37,39–40].

**Scheme 4:**
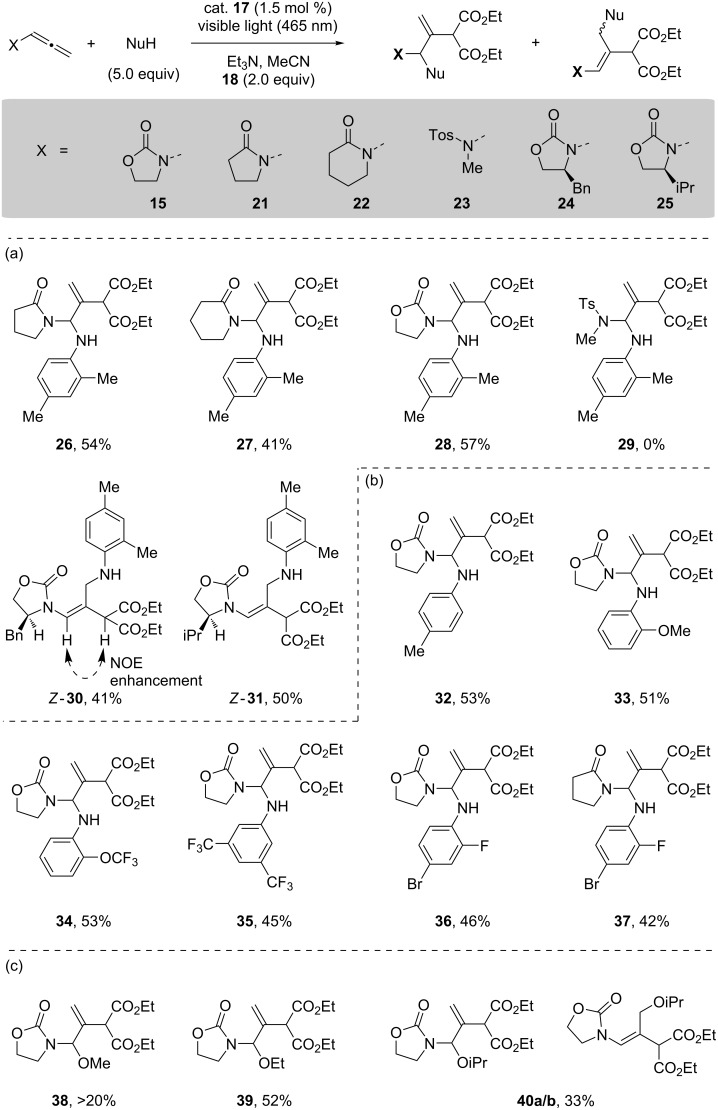
Reaction scope (a) allenamide; (b) arylamine nucleophile; (c) alcohol nucleophile.

An examination of the allenamide unit under these conditions is shown in Scheme 4, and the six allenylamides/sulfonamides (**15**, **21**–**25**) were prepared using known conditions [52–53]. The allenamides derived from pyrrolidinone (**21**), piperidinone (**22**) and oxazolidinone (**15**) with 2,4-dimethylaniline all performed adequately in this reaction giving their *N*,*N*’-allylaminal products **26**, **27** and **28**, respectively. The isolated yields once again reflected the sensitivity of the *N*,*N*’-allylaminal functional group. The aminosulfonyl allenyl **23** failed to provide any discernable product, with only a complex mixture, as identified by ^1^H NMR, being isolated. Significantly, in the ^1^H NMR spectra of this complex mixture we observed complete fragmentation of the sulfonamide unit. Two chiral allenamides (**24** and **25**) were exposed to the photoredox conditions with 2,4-dimethylaniline, and it was observed that the predominant product in each case was *Z*-**30** and *Z*-**31**, respectively, which presumably result from γ-addition of the nucleophile. This was confirmed by ^1^H NMR NOE analysis of *Z*-**30**, where an enhancement between the enamide proton and the methylene proton were observed, as shown in Scheme 4.

Given our interest in the *N*,*N’*-aminal functionality [50], we also explored variation of the arylamine nucleophile. 2,4-Dimethylaniline, *o*-anisole, 2-trifluoromethoxyaniline and 3,5-ditrifluoromethylaniline were all effective in this transformation, giving their labile *N*,*N*’-allylaminal products (**32** to **35**) in moderate to good isolated yields.

4-Bromo-2-fluoroaniline was also examined as a nucleophile, as we had previously shown this to be an effective aniline platform for developing linezolid analogues, and this delivered two *N*,*N*’-allylaminals **36** and **37**, respectively. Masson had previously explored oxygen nucleophiles in their photoredox-catalyzed addition to enamides [48]. Consequently, the use of excess methanol provided the addition product **38**, resulting from α-addition, in a modest isolated yield. The stability of product **38** was marginal, but an improved stability of the *N*,*O’*-allyl product was observed when ethanol was used as the nucleophile giving product **39** in 52% isolated yield. In contrast, isopropanol gave an inseparable mixture of the α- and γ-addition products, **40a/b** in 33% isolated yield. As with the case for the formation of **19**, all of the starting allenes were consumed in each reaction shown in Scheme 4.

**Scheme 5:**
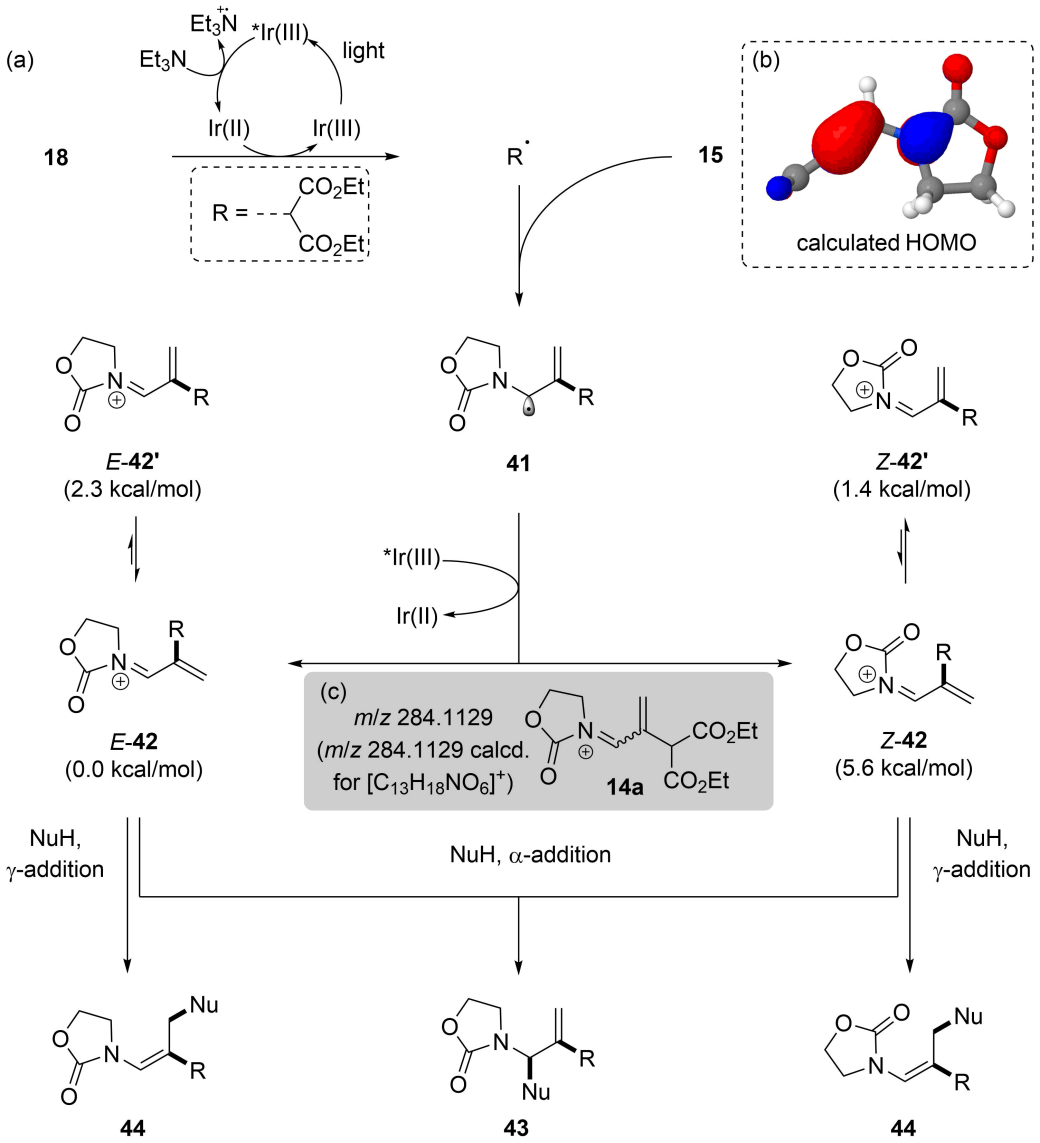
(a) Tentative mechanism for the photoredox-catalyzed formation of the conjugated *N*-acyliminium intermediate; (b) the calculated HOMO [54] for allenamide **15**; (c) the identification of intermediate **14a** by direct sample loop and flow injection HRESIMS analysis.

A tentative mechanism for this transformation is described in Scheme 5a. Excitation of the Ir(III) complex **17** provides *Ir(III) that subsequently undergoes reductive quenching by Et_3_N, delivering Ir(II) [48]. Single electron transfer from Ir(II) to **18** then generates an electrophilic radical R^•^ together with stoichiometric bromide ion; R^•^ subsequently adds to the β-carbon of the allenamide **15**, providing an α-aminoallyl radical **41**. To support this hypothesis the calculated HOMO of allenamide **15** is shown in Scheme 5b, signifying the increased nucleophilicity at the β-carbon [54]. Mechanistically, the formation of the key conjugated *N*-acyliminum **14a** from α-aminoallyl radical **41** is analogous to the photoredox initiated addition of radicals to enamides [46,48–49]. Consequently, the formation of **14a** can results from a SET event between the oxidized Ir(III) [55] and the α-aminoallyl radical **41**; a radical propagation pathway through the reduction of **18** was discounted, as the reaction required continuous irradiation.

To further support this mechanistic hypothesis, we used ESIMS to identify the formation of the conjugated *N*-acyliminium **14a**. The addition of **18** to allenamide **15** under the Ir-catalyzed photoredox conditions in the presence of 4-bromoaniline was monitored by direct sample loop and flow injection analysis by HRESIMS [56–57]. After 5 minutes we observed a peak at *m*/*z* 284.1129 that corresponded satisfactorily to the expected iminium complex **14a** (*m*/*z* 284.1129: calcd for [C_13_H_18_NO_6_]^+^); this peak persisted at 15, 30, 60 and 120 min time intervals, respectively.

Upon the oxidation of **41** two plausible iminium stereoisomers can be formed, *Z*-**42** and *E*-**42**, respectively, with each of these iminium stereoisomers existing in two further conformers designated *E*-**42’** and *Z*-**42’**. DFT calculations [54] were performed on all four of these proposed structures, where it was observed that *Z*-**42** was approx. 6 kcal/mol higher in energy, relative to the most stable isomer *E*-**42**, which was 1–2 kcal/mol lower in energy than *E*-**42’** and *Z*-**42’**, respectively. It is feasible that both *E*-**42** and *Z*-**42’** undergo nucleophilic addition at the α-position giving the observed *N*,*N’*-allylaminal product **43**. Conversely, addition of a nucleophile at the γ-position of *E*-**42** gives the observed *Z*-enamide **44**; and addition at the γ-position of *Z*-**42’** gives the same observed *Z*-enamide **44** after C–N bond rotation.

## Conclusion

In conclusion, in this letter we have demonstrated the first intermolecular addition of an electrophilic radical, generated under photoredox conditions, to an allenamide building block. The addition of the radical occurs at the central carbon of the allene, giving a conjugated *N*-acyliminium intermediate after subsequent oxidation. We have established that the conjugated *N*-acyliminium intermediate can be formed from a broad range of allenamide precursors; additionally, the conjugated *N*-acyliminium intermediate can undergo nucleophilic addition with an arylamine or alcohol nucleophiles at the α- or γ-position, with the regioselectivity of the addition being controlled by steric factors. Significantly, the formation of the key conjugated *N*-acyliminium intermediate using these photoredox conditions can be seen as complementary to the well-developed electrophilic activation modes of allenamides. We are currently examining the full mechanism of this transformation, expanding the scope of substrates that can be used in the radical addition step, and alternative fates for the α-*N*-acyl radical **13** [58–60].

## Supporting Information

File 1 Experimental details, analytical (^1^H NMR, ^13^C NMR) and ESIMS data.
