# Supplementary material for: A complementary approach to conjugated N-acyliminium formation through photoredox-catalyzed intermolecular radical addition to allenamides and allencarbamates
Source: Beilstein J Org Chem. 2020 Aug 12;16:1983–90. doi: 10.3762/bjoc.16.165 (PMC7431758; doi:10.3762/bjoc.16.165)

BEILSTEIN JOURNAL OF ORGANIC CHEMISTRY

Supporting Information

for

A complementary approach to conjugated *N*-acyliminium formation through photoredox-catalyzed intermolecular radical addition to allenamides and allencarbamates

Olusesan K. Koleoso, Matthew Turner, Felix Plasser and Marc C. Kimber

Beilstein J. Org. Chem. **2020,** *16,* 1983–1990. doi:10.3762/bjoc.16.165

Experimental details, analytical (^{1}H NMR, ^{13}C NMR) and ESIMS data

License and Terms: This is a supporting information file under the terms of the Creative Commons Attribution License (http://creativecommons.org/licenses/by/4.0). Please note that the reuse, redistribution and reproduction in particular requires that the authors and source are credited.
The license is subject to the *Beilstein Journal of Organic Chemistry* terms and conditions: (https://www.beilstein-journals.org/bjoc)

Experimental procedures S2–S6

- General information S2
- General photoredox-catalysed addition procedure S2
- Addition product data S2–S6
- DFT calculations for *E*-**42**, *Z*-**42**, *E*-**42’**, *Z*-**42’** and **15** S7–S12
- HRESIMS data for intermediate **14a** S13

Copies of relevant ^{1}H and ^{13}C NMR spectra S14–S31

Experimental

General information

All reactions were carried out using commercially available reagents and solvents throughout without further purification, except dichloromethane (CH_2Cl_2) and triethylamine (Et_3N) which were dried using 4 Å molecular sieve. DMSO and acetonitrile were purchased dry from commercial suppliers. Light petroleum refers to the fraction with bp 40–60 °C. Thin-layer chromatography was carried out on Merck Kieselgel 60 GF254 aluminum foil backed plates. The plates were visualized under UV light and vallinin stain. Flash chromatography was carried out using Merck Kieselgel 60H silica or Matrix silica 60, with the eluent as specified in the individual syntheses. IR spectra were recorded using a Perkin Elmer FTIR Spectrometer (Paragon 100) as solutions in CH_2Cl_2, unless otherwise stated. ^{1}H & ^{13}C NMR spectra were recorded using a Bruker 400 MHz NMR machine and a JEOL ECS-400 MHz NMR machine; chemical shifts were quoted in ppm and coupling constants, *J*, were quoted in Hz; *d*-chloroform was used throughout unless otherwise stated. Spectra were calibrated to residual solvent peaks. High-resolution mass spectra were carried out on a Thermofisher exactive (orbi) resolution mass spectrometer. Allenamides **15**, **21**, **22**, **23**, **24** and **25** were prepared using literature conditions,[1] and ^{1}H & ^{13}C NMR spectra are provided on pages S14 to S31.

General procedure for the photoredox-catalysed radical addition to allenamides

An oven dried 50 mL three neck round bottom flask was filled with argon gas and charged with iridium photocatalyst **17** (1.5 mol %), the allenamide (1.00 equiv) and anhydrous acetonitrile (≈0.1 M) and seal with a rubber septum in the middle neck and the other two necks closed with glass stoppers. The flask was purged again with argon while one neck was opened. The middle neck was then fitted with an argon filled balloon. Diethyl bromomalonate **18** (2.00 equiv), trimethylamine (2.00 equiv) and the nucleophile (5.00 equiv) were added to the solution and placed under an argon atmosphere. The flask was then irradiated with a Kessil blue light (λ = 467 nm) for 3 h with stirring at room temperature. After the reaction was complete as determined by TLC, the solvent was removed under a reduced pressure. The crude product was then purified by flash chromatography on silica gel to afford the following products:

Diethyl 2-(3-((2,4-dimethylphenyl)amino)-3-(2-oxopyrrolidin-1-yl)prop-1-en-2 yl)malonate (26).

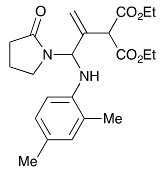

The crude product was purified by flash chromatography on silica gel (hexane / EtOAc 4:1) to afford the product **26** as a pale yellow oil (106 mg, 54%). ^{1}H-NMR (400 MHz, $CDCl_3$) δ 6.87 – 6.38 (m, 2H), 6.52 (d, *J* = 8.4 Hz, 1H), 6.20 (d, *J* = 10.0 Hz, 1H), 5.42 (d, *J* = 2.0 Hz, 1H), 5.34 (d, *J* = 1.6 Hz, 1H), 4.79 (d, *J* = 10.4 Hz, 1H), 4.31 (s, 1H), 4.29 - 4.17 (m, 2H), 4.12 - 4.02 (m, 2H), 3.28 -3.25 (m, 1H), 3.06 (dd, *J* = 8.4, 16.0 Hz, 1H), 2.46 - 2.37 (2H),

2.20 (s, 3H), 2.09 (s, 3H), 2.00 - 1.85 (m, 2H), 1.29 (t, *J* = 7.2 Hz, 3H), 1.15 (t, *J* = 7.2 Hz, 3H) ppm; ^{13}C-NMR (100 MHz, $CDCl_3$) δ 175.9, 169.2, 168.1, 140.2, 137.7, 131.2, 127.7, 122.7, 117.8, 111.0, 62.7, 62.1, 55.4,

(1) T. W. Bousfield and M. C. Kimber, *Tetrahedron Lett.* 2015, *56*, 350.

41.5, 31.4, 20.5, 18.0, 17.5, 14.1 ppm; IR υ (cm^{-1}) 3018, 2941, 1727, 1677, 1420, 1176, 1096, 1035; HRMS [M+H] calculated for $C_{22}H_{31}N_2O_5$ 425.2047, found 425.2068.

Diethyl 2-(3-((2,4-dimethylphenyl)amino)-3-(2-oxopiperidin-1-yl)prop-1-en-2 yl)malonate (27).

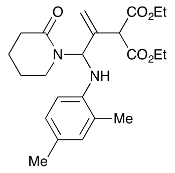

The crude product was purified by flash chromatography on silica gel (hexane / EtOAc 4:1) to afford the product **27** as a pale yellow liquid (86 mg, 41%). ^{1}H-NMR (400 MHz, $CDCl_3$) δ 6.91 – 6.86 (m, 2H), 6.77 (d, *J* = 10.4 Hz, 1H), 6.50 (d, *J* = 8.8 Hz, 1H), 5.39 (d, *J* = 2.0 Hz, 1H), 5.31 (d, *J* = 2.0 Hz, 1H), 4.92 (d, *J* = 10.4 Hz, 1H), 4.33 (s, 1H), 4.32 - 3.99 (m, 3H), 4.05 – 4.01 (m, 2H), 3.16 - 3.11 (m, 1H), 2.92 - 2.89 (m, 1H), 2.48 - 2.42 (m, 2H), 2.20 (s, 3H), 2.09 (s, 3H), 1.74 - 1.66 (m, 5H), 1.29 (t, *J* = 7.2 Hz, 3H), 1.14 (t, *J* = 7.2 Hz, 3H) ppm; ^{13}C-NMR (100 MHz, $CDCl_3$) δ 170.7, 169.5, 168.2, 140.3, 137.6, 131.3, 127.6, 122.6, 117.5, 111.1, 64.0, 62.4, 55.4, 40.6, 32.4, 23.0, 20.6, 17.6, 14.1 ppm; IR υ (cm^{-1}) 3018, 2984, 1731, 1660, 1216, 1371, 1033; HRMS [M+Na] calculated for $C_{23}H_{32}N_2O_5Na$ 439.2203, found 439.2217.

Diethyl 2-(3-((2,4-dimethylphenyl)amino)-3-(2-oxooxazolidin-3-yl)prop-1-en-2-yl)malonate (28).

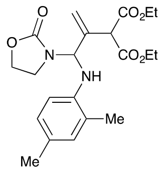

The crude product was purified by flash chromatography on silica gel (hexane / EtOAc 3:7) to afford the product **28** as a colourless oil (109 mg, 57%). ^{1}H-NMR (400Mhz, $CDCl_3$) δ 7.92 - 6.87 (m, 2H), 6.64 - 6.52 (m, 1H), 6.04 (d, *J* = 10.0 Hz, 1H), 5.49 (d, *J* = 2.0 Hz, 1H), 5.47 (d, *J* = 2.0 Hz, 1H), 4.94 (d, *J* = 9.6 Hz, 1H), 4.34 - 4.01 (m, 6H), 3.82 – 3.81 (m, 1H), 3.47 – 3.43 (m, 1H), 3.30 (q, *J* = 8.8 Hz, 1H), 2.22 (s, 3H), 2.11 (s, 3H), 1.30 (t, *J* = 7.2 Hz, 3H), 1.15 (t, *J* = 7.2 Hz, 3H); ^{13}C-NMR (100Mhz, $CDCl_3$) δ 169.3, 168.7, 167.8, 158.3, 139.8, 137.7, 131.4, 131.1, 127.9, 127.3, 122.8, 118.7, 111.0, 109.9, 64.7, 62.7, 62.3, 58.2, 55.6, 44.4, 42.2, 39.2, 20.5, 17.7, 17.6, 14.1. IR υ (cm^{-1}) 3342, 2969, 2930, 2881, 1738, 1517, 1466, 1128, 1034, 950; HRMS [M+H] calculated for $C_{21}H_{28}N_2O_6$ 405.2020, found 405.2017.

Diethyl (*S,Z*)-2-(1-(4-benzyl-2-oxooxazolidin-3-yl)-3-((2,4-dimethylphenyl)amino)prop-1-en-2-yl) malonate (*Z*-30).

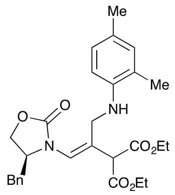

The crude product was purified by flash chromatography on silica gel (hexane / EtOAc 7:3) to afford the product *Z*-**30** as a pale yellow liquid (97 mg, 41%). ^{1}H-NMR (400 MHz, $CDCl_3$) δ 7.28 - 7.21 (m, 3H), 7.10 – 7.08 (m, 2H), 6.91 (d, *J* = 8.0 Hz, 2H), 6.88 (s, 1H), 6.51 (d, *J* = 8.4 Hz, 1H), 6.38 (s, 1H), 4.39 - 4.31 (m, 1H), 4.28 (s, 1H), 4.22 - 4.05 (m, 6H), 3.93 (d, *J* = 8.8 Hz, 2H), 3.17 (dd, *J* = 4.0, 12.8 Hz, 1H), 2.68 (d, *J* = 10.0, 13.6 Hz, 1H), 2.22 (s, 3H), 2.09 (s, 3H), 1.60 (s, 1H), 1.27 - 1.19 (m, 6H) ppm; ^{13}C-NMR (100 MHz, $CDCl_3$) δ 168.4, 156.4, 143.8, 135.1, 131.1, 129.2, 127.1, 126.1, 123.3, 110.0, 77.1, 66.7, 62.1, 57.9, 56.5, 43.8, 38.4, 20.4, 17.5, 14.1 ppm; IR υ (cm^{-1}) 3031, 2985, 1756, 1731, 1516, 1302, 1266, 1032; HRMS [M+H] calculated for $C_{28}H_{35}N_2O_6$ 495.2490, found 495.2487.

Diethyl (*S,Z*)-2-(3-((2,4-dimethylphenyl)amino)-1-(4-isopropyl-2-oxooxazolidin-3-yl)prop-1-en-2-yl) malonate (*Z*-31).

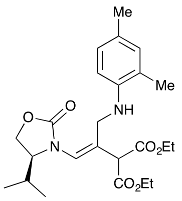

The crude product was purified by flash chromatography on silica gel (hexane / EtOAc 4:1) to afford the product *Z*-**31** as a pale yellow liquid (107 mg, 50%). ^{1}H-NMR (400 MHz, $CDCl_3$) δ 6.90 - 6.86 (m, 2H), 6.47 (d, *J* = 7.8 Hz, 1H), 6.24 (s, 1H), 4.29 - 4.22 (m, 2H), 4.20 - 4.04 (m, 5H), 4.01 - 3.94 (m, 1H), 3.89 (d, *J* = 13.6 Hz, 1H), 3.79 (d, *J* = 13.6 Hz, 1H), 2.20 (s, 3H), 2.08 (s, 3H), 2.10 - 2.07 (1H), 1.27 - 1.17 (m, 6H), 0.87 (t, *J* = 6.6 Hz, 6H) ppm; ^{13}C-NMR (100 MHz, $CDCl_3$) δ 168.4, 168.3, 156.7, 143.9, 131.0, 127.2, 126.65, 126.2, 124.3, 123.1, 110.0, 63.9, 62.0, 61.6, 57.0, 43.8, 29.4, 20.4, 17.9, 17.4, 15.1, 14.0 ppm; IR *υ* (cm^{-1}) 3157, 2982, 2253, 1746, 1664, 1467, 1261, 1095, 1033; HRMS [M+H] calculated for $C_{24}H_{35}N_2O_6$ 447.2490, found 447.2510.

Diethyl 2-(3-(2-oxooxazolidin-3-yl)-3-(*p*-tolylamino)prop-1-en-2-yl)malonate (32)

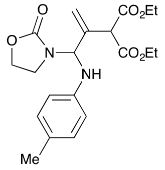

The crude product was purified by flash chromatography on silica gel (hexane / EtOAc 3:7) to afford the product **32** as a pale yellow oil (98 mg, 53%). ^{1}H-NMR (400 MHz, $CDCl_3$) δ 6.98 (d, *J* = 8.4 Hz, 2H), 6.60 (d, *J* = 8.4 Hz, 2H), 6.52 (d, *J* = 8.2 Hz, 1H), 5.96 (d, *J* = 9.2 Hz, 1H), 5.47 (d, *J* = 2.0, Hz, 1H), 5.45 (d, *J* = 2.0 Hz, 1H), 4.93 (d, *J* = 9.2 Hz, 1H), 4.31 - 4.02 (m, 8H), 3.79 (s, 1H), 3.46 - 3.34 (m, 2H), 2.21 (s, 3H), 1.31 - 1.20 (m, 6H) ppm; ^{13}C-NMR (100 MHz, $CDCl_3$) δ 169.0, 168.6, 167.7, 158.3, 141.9, 137.6, 130.1, 128.6, 118.5, 113.8, 113.3, 65.3, 62.3, 62.0, 55.5, 39.5, 31.7, 22.7, 20.5, 14.0 ppm; IR *υ* (cm^{-1}) 3328, 2969, 2931, 1750, 1684, 1595, 1466, 1378, 1367, 1306, 1127, 1107, 950; HRMS [M+H] calculated for $C_{20}H_{27}N_2O_6$ 391.1864, found 391.1860.

Diethyl 2-(3-((2-methoxyphenyl)amino)-3-(2-oxooxazolidin-3-yl)prop-1-en-2-yl)malonate (33).

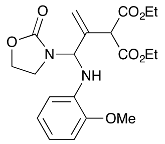

The crude product was purified by flash chromatography on silica gel (hexane / EtOAc 3:7) to afford the product **34** as a pale yellow oil (99 mg, 51%). ^{1}H-NMR (400Mhz, $CDCl_3$) δ 6.84 - 6.73 (m, 4H), 5.98 (d, *J* = 8.8 Hz, 1H), 5.52 (d, *J* = 2.0 Hz, 2H), 5.50 (m, 1H), 5.16 (d, *J* = 9.6 Hz, 1H), 4.26 - 4.12 (m, 7H), 3.81 (s, 3H), 3.44 – 3.41 (m, 1H), 3.33 - 3.26 (m, 1H), 1.26 (t, *J* = 7.2 Hz, 3H), 1.24 (t, *J* = 7.2 Hz, 3H); ^{13}C-NMR (100Mhz, $CDCl_3$) δ 168.2, 167.8, 158.4, 147.1, 137.4, 134.0, 121.8, 118.8, 118.0, 111.7, 110.0, 65.0, 62.5, 62.2, 55.7, 55.1, 39.2, 14.1; IR *υ* (cm^{-1}) 3331, 2969, 2931, 2882, 1739, 1466, 1378, 1367, 1340, 1305, 1159, 1127, 1031, 950. HRMS [M+H] calculated for $C_{20}H_{26}N_2O_7$ 407.1812, found 407.1811.

Diethyl 2-(3-(2-oxooxazolidin-3-yl)-3-((2-(trifluoromethoxy)phenyl)amino)prop-1-en-2-yl)malonate (34).

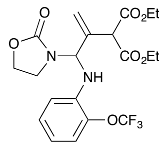

The crude product was purified by flash chromatography on silica gel (hexane / EtOAc 3:7) to afford the product **35** as a pale yellow liquid (117 mg, 53%). ^{1}H-NMR (400Mhz, $CDCl_3$) δ 7.16 (t, *J* = 7.6 Hz, 2H), 6.89 (d, *J* = 7.4 Hz, 1H), 6.76 (td, *J* = 7.7, 1.4 Hz, 1H), 6.07 (d, *J* = 9.2 Hz, 1H), 5.66 (d, *J* = 9.6 Hz, 1H), 5.51 (d, *J* = 2.0 Hz, 2H), 5.49 (d, *J* = 2.0 Hz, 1H), 4.29 – 4.26 (m, 3H), 4.21 – 4.18 (m, 2H), 4.09 – 4.05 (m, 2H), 3.50 – 3.46 (m, 1H), 3.31 (q, *J* =

9.2 Hz, 1H), 1.28 (t, *J* = 7.2 Hz, 3H), 1.15 (t, *J* = 7.2 Hz, 3H); ^{13}C-NMR (100Mhz, $CDCl_3$) δ 168.9, 167.5, 158.3, 136.9, 136.8, 136.5, 128.4, 121.4, 119.2, 118.8, 113.4, 64.1, 62.3, 55.4, 39.0, 14.00. IR *υ* (cm^{-1}) 3337, 2969, 2931, 1739, 1613, 1466, 1378, 1308, 1159, 1128, 1107, 1034. HRMS [M+H] calculated for $C_{20}H_{23}N_2O_7F_3$ 461.1530, found 461.1532.

Diethyl 2-(3-((3,5-bis(trifluoromethyl)phenyl)amino)-3-(2-oxooxazolidin-3-yl)prop-1-en-2-yl)malonate (35).

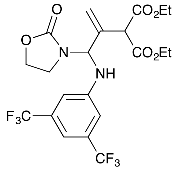

The crude product was purified by flash chromatography on silica gel (hexane / EtOAc 3:7) to afford the product **36** as a pale yellow oil (113 mg, 45%). ^{1}H-NMR (400Mhz, $CDCl_3$) δ 7.25 - 7.23 (m, 1H), 7.11 (s, 2H), 6.17 (d, *J* = 8.8 Hz, 1H), 6.07 (d, *J* = 8.8 Hz, 1H), 5.51 (d, *J* = 2.0 Hz, 1H), 5.50 (d, *J* = 2.0 Hz, 1H), 4.37 - 4.18 (m, 4H), 4.03 - 3.97 (m, 2H), 3.52 - 3.39 (m, 2H), 1.31 (t, *J* = 7.2 Hz, 3H), 1.11 (t, *J* = 7.0 Hz, 3H); ^{13}C-NMR (100Mhz, $CDCl_3$) δ 169.7, 167.1, 158.2, 145.6, 136.4, 132.8 (q, *J* = 32.4 Hz), 124.7, 122.0, 120.3, 113.2, 112.3, 64.3, 62.7, 62.5, 55.9, 39.5, 13.9. IR *υ* (cm^{-1}) 3332, 2969, 2931, 1741, 1622, 1466, 1378, 1107, 1036. HRMS [M+Na] calculated for $C_{21}H_{22}N_2O_6F_6$ 535.1274, found 535.1276.

Diethyl 2-(3-((4-bromo-2-fluorophenyl)amino)-3-(2-oxooxazolidin-3-yl)prop-1-en-2- yl)malonate (36).

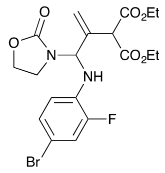

The crude product was purified by flash chromatography on silica gel (hexane / EtOAc 7:3) to afford the product **37** as a pale yellow liquid (102 mg, 46%). ^{1}H-NMR (400 MHz, $CDCl_3$) δ 7.16 - 7.09 (m, 2H), 6.78 - 6.74 (m, 1H), 6.01 (d, *J* = 9.2 Hz, 1H), 5.51 (d, *J* = 2.0 Hz, 1H), 5.50 (d, *J* = 2.0 Hz, 1H), 5.44 (dd, *J* = 6.0, 8.8 Hz, 1H), 4.30 - 4.21 (m, 5H), 4.12 - 4.05 (m, 2H), 3.47 (dd, *J* = 6.0, 8.8 Hz, 1H), 3.35 (q, *J* = 8.8 Hz, 1H), 1.29 (t, *J* = 7.2 Hz, 3H), 1.17 (t,

J = 7.2 Hz, 3H) ppm; ^{13}C-NMR (100 MHz, $CDCl_3$) δ 168.9, 167.3, 160.1, 158.3, 136.7, 132.1, 128.1, 119.5, 118.5, 118.3, 114.8, 109.8, 64.3, 62.4, 55.6, 39.1, 14.0 ppm; IR *υ* (cm^{-1}) 3155, 2985, 1793, 1748, 1614, 114, 1194, 1096, 1034; HRMS [M+Na] calculated for $C_{19}H22_{Br}N_2O_6Na$ 495.0537, found 495.0559.

Diethyl 2-(3-((4-bromo-2-fluorophenyl)amino)-3-(2-oxopyrrolidin-1-yl)prop-1-en-2- yl)malonate (37).

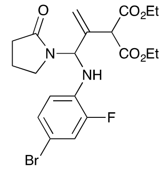

The crude product was purified by flash chromatography on silica gel (hexane / EtOAc 4:1) to afford the product **38** as a pale yellow liquid (97 mg, 42%). ^{1}H-NMR (400 MHz, $CDCl_3$) δ 7.14 (d, *J* = 2.1 Hz, 1H), 7.12 - 7.06 (m, 2H), 6.65 (t, *J* = 8.9 Hz, 1H), 6.18 (d, *J* = 9.9 Hz, 1H), 5.46 (d, *J* = 2.0 Hz, 1H), 5.37 (d, *J* = 2.0 Hz, 1H), 5.33 – 5.29 (m, 1H), 4.30 - 4.16 (m, 3H),4.10 (q, *J* = 7.1 Hz, 3H), 3.27 - 3.25 (m, 1H), 3.12 - 3.07 (m, 1H), 2.44 - 2.36 (m, 2H), 1.99 - 1.90 (m, 2H), 1.28 (t, *J* = 7.2 Hz, 3H), 1.20 (t, *J* = 7.0 Hz, 3H) ppm; ^{13}C-NMR (100 MHz, $CDCl_3$) δ 176.1, 168.8, 167.6, 152.6, 150.1, 136.6, 132.3, 127.9, 118.7, 118.4, 118.2, 114.6, 109.3, 62.3, 61.9, 55.3, 41.5, 31.2, 18.1, 14.1 ppm; IR *υ* (cm^{-1}) 3054, 728, 1686, 1614, 1421, 1265, 1154, 1035; HRMS [M+Na] calculated for $C_{20}H_{24}BrFN_2O_5Na$ 493.0745, found 493.0767.

Diethyl 2-(3-ethoxy-3-(2-oxooxazolidin-3-yl)prop-1-en-2-yl)malonate (39).

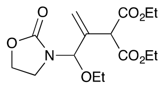

The crude product was purified by flash chromatography on silica gel (hexane / EtOAc 2:1) to afford the product **40** as a pale-yellow oil (65 mg, 52%). ^{1}H-NMR (400 MHz, $CDCl_3$) δ 5.61 (d, *J* = 1.6 Hz, 1H), 5.56 (m, 1H), 5.43 - 5.41 (m, 1H), 4.39 - 4.25 (m, 2H), 4.22 - 4.15 (m, 4H), 4.05 (s, 1H), 3.61 - 3.47 (m, 2H), 3.43 (dd, *J* = 8.8, 7.4 Hz, 2H), 1.31 – 1.25 (m, 6H), 1.19 (t, *J* = 7.2 Hz, 3H) ppm; ^{13}C-NMR (100 MHz, $CDCl_3$) δ 168.5, 167.6, 167.5, 158.6, 136.9, 118.5, 83.1, 64.3, 63.6, 62.7, 62.0, 61.9, 54.5, 39.0, 14.9, 14.1 ppm; IR *υ* (cm^{-1}) 3329, 2969, 2931, 2882, 2657, 1745, 1466, 1378, 1305, 1159, 1127, 1107; HRMS [M+Na] calculated for $C_{15}H_{23}NO_7Na$ 352.1367, found 352.1367.

Diethyl 2-(3-isopropoxy-3-(2-oxooxazolidin-3-yl)prop-1-en-2-yl)malonate (40a) and diethyl (*S,Z*)-2-(3-(isopropoxy)-1-(2-oxooxazolidin-3-yl)prop-1-en-2-yl)malonate (*Z*-40b).

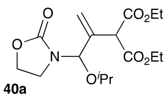

40a

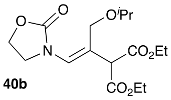

40b

The crude product was purified by flash chromatography on silica gel (hexane / EtOAc 2:1) to afford the product **40a** and **40b** as an inseparable, 1:3 mixture (55 mg, 33%). **40a** ^{1}H-NMR (400 MHz, $CDCl_3$) δ 6.27 (s, 1H), 5.43 (s, 1H), 5.39 (s, 1H), 4.57 (dd, *J* = 4.4, 10.0 Hz, 1H), 4.25 – 4.17 (m, 10H), 3.92 – 3.88 (m, 1H), 3.82 – 3.78 (m, 1H), 3.44 – 3.40 (m, 1H), 3.38 – 3.32 (m, 1H), 2.87 – 2.79 (m, 1H), 1.27 – 1.21 (m, 12H) ppm; **40b** ^{1}H-NMR (400 MHz, $CDCl_3$) δ 6.25 (s, 1H), 4.42 - 4.40 (m, 2H), 4.25 – 4.17 (m, 4H), 4.90 – 3.84 (m,

2H), 2.87 – 2.79 (m, 1H), 2.40 (s, 2H), 1.27 – 1.21 (m, 12H) ppm; ^{13}C-NMR (100 MHz, $CDCl_3$) δ 168.1, 167.9, 167.6, 158.2, 157.1, 138.4, 128.3, 126.5, 123.3, 121.7, 118.9, 62.7, 62.3, 62.1, 57.1, 56.4, 56.0, 54.6, 46.3, 45.9, 41.3, 20.0, 28.0, 14.1 ppm; IR *υ* (cm^{-1}) 3055, 2987, 2306, 1755, 1667, 1265, 1155, 1035; HRMS [M+Na] calculated for C_{16}H25NO_7Na 366.1523, found 366.1511.

Table S1. Calculated relative energies for *Z*-**42**, *Z*-**42'**, *E*-**42** and *E*-**42'**

Structure	Relative Gibbs free energy (kJ/mol)	Relative Gibbs free energy (kcal/mol)
Z-**42**	5.64	1.35
E-**42**	9.81	2.34
Z-**42'**	23.59	5.63
E-**42'**	0.00	0.00

Computational Details

Geometry optimisations were performed using density functional theory at the PBEh-3c level of theory.[2] Solvation in acetonitrile was modelled using a conductor-like polarisable continuum model[3] considering a dielectric constant of 35.688. Vibrational analyses were performed verifying that all four optimised structures are true minima. The values reported are Gibbs free energies computed for 298 K. All computations were performed using Q-Chem 5.1.[4]

2 S. Grimme, J. G. Brandenburg, C. Bannwarth and A. Hansen, *J. Chem. Phys.*, 2015, **143**, 054107.

3 V. Barone and M. Cossi, *J. Phys. Chem. A*, 1998, **102**, 1995–2001.

4 Y. Shao, Z. Gan, E. Epifanovsky, A. T. B. Gilbert, M. Wormit, J. Kussmann, A. W. Lange, A. Behn, J. Deng, X. Feng, D. Ghosh, M. Goldey, P. R. Horn, L. D. Jacobson, I. Kaliman, R. Z. Khaliullin, T. Kus, A. Landau, J. Liu, E. I. Proynov, Y. M. Rhee, R. M. Richard, M. A. Rohrdanz, R. P. Steele, E. J. Sundstrom, H. L. Woodcock, P. M. Zimmerman, D. Zuev, B. Albrecht, E. Alguire, B. Austin, G. J. O. Beran, Y. A. Bernard, E. Berquist, K. Brandhorst, K. B. Bravaya, S. T. Brown, D. Casanova, C. M. Chang, Y. Chen, S. H. Chien, K. D. Closser, D. L. Crittenden, M. Diedenhofen, R. A. Distasio, H. Do, A. D. Dutoi, R. G. Edgar, S. Fatehi, L. Fusti-Molnar, A. Ghysels, A. Golubeva-Zadorozhnaya, J. Gomes, M. W. D. Hanson-Heine, P. H. P. Harbach, A. W. Hauser, E. G. Hohenstein, Z. C. Holden, T. C. Jagau, H. Ji, B. Kaduk, K. Khistyaev, J. Kim, J. Kim, R. A. King, P. Klunzinger, D. Kosenkov, T. Kowalczyk, C. M. Krauter, K. U. Lao, A. D. Laurent, K. V. Lawler, S. V. Levchenko, C. Y. Lin, F. Liu, E. Livshits, R. C. Lochan, A. Luenser, P. Manohar, S. F. Manzer, S. P. Mao, N. Mardirossian, A. V. Marenich, S. A. Maurer, N. J. Mayhall, E. Neuscamman, C. M. Oana, R. Olivares-Amaya, D. P. Oneill, J. A. Parkhill, T. M. Perrine, R. Peverati, A. Prociuk, D. R. Rehn, E. Rosta, N. J. Russ, S. M. Sharada, S. Sharma, D. W. Small, A. Sodt, T. Stein, D. Stück, Y. C. Su, A. J. W. Thom, T. Tsuchimochi, V. Vanovschi, L. Vogt, O. Vydrov, T. Wang, M. A. Watson, J. Wenzel, A. White, C. F. Williams, J. Yang, S. Yeganeh, S. R. Yost, Z. Q. You, I. Y. Zhang, X. Zhang, Y. Zhao, B. R. Brooks, G. K. L. Chan, D. M. Chipman, C. J. Cramer, W. A. Goddard, M. S. Gordon, W. J. Hehre, A. Klamt, H. F. Schaefer, M. W. Schmidt, C. D. Sherrill, D. G. Truhlar, A. Warshel, X. Xu, A. Aspuru-Guzik, R. Baer, A. T. Bell, N. A. Besley, J. Da Chai, A. Dreuw, B. D. Dunietz, T. R. Furlani, S. R. Gwaltney, C. P. Hsu, Y. Jung, J. Kong, D. S. Lambrecht, W. Liang, C. Ochsenfeld, V. A. Rassolov, L. V. Slipchenko, J. E. Subotnik, T. Van Voorhis, J. M. Herbert, A. I. Krylov, P. M. W. Gill and M. Head-Gordon, *Mol. Phys.*, 2015, **113**, 184–215.

Geometries and total energies of the optimised structures

Table S2: Total SCF energy (a.u.) and molecular coordinates (Å) of *Z*-**42** optimised at the PBEh-3c level of theory in acetonitrile.

Total energy: -1010.005493475

C	-2.54130	-2.41098	0.73128
C	-3.70220	-2.69243	1.66889
N	-2.02369	-1.16959	1.30877
H	-2.85019	-2.25978	-0.29899
H	-1.76847	-3.17816	0.78175
O	-3.32561	-2.03234	2.88972
H	-3.83471	-3.74942	1.87764
H	-4.63765	-2.26105	1.31523
C	-2.42214	-1.10431	2.69801
O	-1.97044	-0.38053	3.51976
C	-1.17409	-0.39352	0.71372
C	-0.78037	0.94135	1.13543
H	-0.86127	-0.73580	-0.26944
C	-1.69139	1.84206	1.49502
H	-2.74680	1.61292	1.54841
H	-1.40081	2.86297	1.70329
C	0.68131	1.24713	0.97765
C	1.11599	1.47704	-0.47244
C	1.45637	0.06619	1.52355
H	0.92270	2.16169	1.52931
O	2.63088	0.36762	1.98930
O	0.97013	-1.04427	1.49725
O	2.15779	1.07382	-0.91456
C	3.46978	-0.70280	2.48659
O	0.22065	2.17502	-1.12681
C	0.49684	2.52257	-2.50092
C	4.75184	-0.08421	2.98029
H	3.65375	-1.41146	1.67795
H	2.94420	-1.22298	3.28861
H	5.28715	0.42549	2.17981
H	5.39694	-0.87344	3.36483
H	4.57001	0.62285	3.78912
C	-0.70449	3.26038	-3.03451
H	0.68784	1.61228	-3.07135
H	1.39332	3.14370	-2.53779
H	-1.59845	2.63732	-3.01779
H	-0.51398	3.54337	-4.06923
H	-0.90102	4.17142	-2.46973

Table S3: Total SCF energy (a.u.) and molecular coordinates (Å) of *E*-**42** optimised at the PBEh-3c level of theory in acetonitrile.

Total energy: -1010.004098696

C	-5.18639	2.70167	0.94974
C	-4.01399	1.91525	1.52263
N	-5.66708	1.78478	-0.07391
H	-4.86138	3.63087	0.48219
H	-5.96178	2.90725	1.68621
O	-3.66426	0.98402	0.48422
H	-4.28862	1.34937	2.41111
H	-3.14987	2.53892	1.72822
C	-4.62655	0.81955	-0.37906
O	-4.67834	0.03886	-1.26885
C	-6.82118	1.69603	-0.65008
C	-7.96107	2.54922	-0.47213
H	-6.92397	0.84689	-1.32105
C	-7.84131	3.84297	-0.13523
H	-6.89050	4.32672	0.03094
H	-8.70672	4.48758	-0.06446
C	-9.29278	1.90945	-0.81066
C	-9.80482	2.28204	-2.19756
C	-10.38450	2.21591	0.20887
H	-9.18420	0.81968	-0.80020
O	-9.92252	2.16321	1.43586
O	-11.52533	2.42751	-0.10384
O	-10.17105	1.46948	-3.00240
C	-10.84314	2.38994	2.52367
O	-9.79855	3.58325	-2.37684
C	-10.28422	4.10385	-3.62941
C	-10.05803	2.30541	3.80798
H	-11.63180	1.63643	2.49043
H	-11.30369	3.37208	2.40510
H	-9.60633	1.32235	3.93900
H	-10.73172	2.47801	4.64665
H	-9.27325	3.06055	3.84848
C	-10.17956	5.60686	-3.57116
H	-11.31784	3.78508	-3.77468
H	-9.68370	3.69697	-4.44482
H	-10.78801	6.02050	-2.76715
H	-10.53954	6.02412	-4.51105
H	-9.14861	5.93280	-3.43407

Table S4: Total SCF energy (a.u.) and molecular coordinates (Å) of *Z*-**42'** optimised at the PBEh-3c level of theory in acetonitrile.

Total energy: -1010.000860625

C	-3.42502	-0.01284	-0.50710
C	-4.44758	-0.96626	0.08497
N	-2.27321	-0.28726	0.36068
H	-3.71584	1.03166	-0.39557
H	-3.18901	-0.22859	-1.54492
O	-4.02659	-1.10481	1.45443
H	-4.42603	-1.94793	-0.38608
H	-5.45724	-0.56808	0.07606
C	-2.76967	-0.79131	1.61622
O	-2.15351	-0.84143	2.62934
C	-1.05167	0.02766	0.05109
C	0.19040	-0.27227	0.69931
H	-0.97637	0.60177	-0.86664
C	1.17802	0.57569	0.35229
H	1.02202	1.36677	-0.36926
H	2.16376	0.52028	0.79501
C	0.42588	-1.44358	1.62546
C	0.51961	-1.10361	3.10590
C	1.72189	-2.13475	1.20507
H	-0.36619	-2.18542	1.51131
O	1.53598	-2.82330	0.10149
O	2.76117	-2.03030	1.79939
O	0.51202	-1.95640	3.95201
C	2.67249	-3.48777	-0.48815
O	0.63494	0.18553	3.32285
C	0.72367	0.63971	4.68579
C	2.19372	-4.19663	-1.72976
H	3.09183	-4.19227	0.23193
H	3.43746	-2.74627	-0.72537
H	1.44061	-4.94904	-1.49679
H	3.03824	-4.70259	-2.19645
H	1.77892	-3.49869	-2.45673
C	0.79754	2.14583	4.66095
H	1.61023	0.20778	5.15351
H	-0.15312	0.29672	5.23787
H	1.67398	2.49581	4.11583
H	0.87077	2.51473	5.68342
H	-0.09309	2.58365	4.21044

Table S5: Total SCF energy (a.u.) and molecular coordinates (Å) of ***E*-42'** optimised at the PBEh-3c level of theory in acetonitrile.

Total energy: -1010.007257995

C	-5.04321	2.03447	1.02984
C	-3.93423	1.02162	1.29104
N	-5.43308	1.68837	-0.33467
H	-4.67564	3.06005	1.05069
H	-5.86834	1.91501	1.72627
O	-3.51835	0.58937	-0.01335
H	-4.29130	0.15279	1.84110
H	-3.07882	1.45744	1.79760
C	-4.40025	0.86667	-0.93233
O	-4.38381	0.53040	-2.06957
C	-6.47438	2.00068	-1.04346
C	-7.60694	2.80828	-0.72314
H	-6.46999	1.56073	-2.03563
C	-8.56260	2.78074	-1.67514
H	-8.43586	2.21260	-2.58739
H	-9.49766	3.31523	-1.57771
C	-7.77113	3.58929	0.55843
C	-8.59547	2.81862	1.58509
C	-8.39391	4.97126	0.37369
H	-6.79826	3.79727	1.00784
O	-7.99188	5.54260	-0.73565
O	-9.10886	5.46650	1.20255
O	-8.18830	2.52150	2.67605
C	-8.44245	6.88573	-1.01586
O	-9.78118	2.52480	1.10616
C	-10.70066	1.79912	1.94766
C	-7.87527	7.28573	-2.35392
H	-8.09717	7.55029	-0.22223
H	-9.53335	6.90314	-1.02677
H	-6.78562	7.27576	-2.34799
H	-8.19895	8.29982	-2.58608
H	-8.22808	6.63164	-3.15103
C	-11.98523	1.62441	1.17841
H	-10.86529	2.36174	2.86816
H	-10.26087	0.83543	2.20989
H	-12.43336	2.58436	0.92300
H	-12.69606	1.07833	1.79772
H	-11.83209	1.05354	0.26275

Table S6: Total SCF energy (a.u.) and molecular coordinates (Å) of **15** optimised at the PBEh-3c level of theory in acetonitrile.

Total energy: -437.072505111

C	-5.42328	2.92709	0.40245
C	-4.01139	2.41991	0.69632
N	-5.88643	1.90416	-0.50281
H	-5.43402	3.90794	-0.08013
H	-6.03935	2.97782	1.30016
O	-3.73734	1.47288	-0.34224
H	-3.95525	1.91154	1.65937
H	-3.26136	3.20635	0.66300
C	-4.86688	1.13555	-0.97241
O	-4.91989	0.27955	-1.81792
C	-7.17604	1.85046	-1.01641
C	-8.13074	2.67480	-0.67159
C	-9.09985	3.47792	-0.34175
H	-7.35549	1.06157	-1.73707
H	-9.28321	4.40054	-0.88224
H	-9.76533	3.25763	0.48634

Figure S1: HRESIMS data for the detection of iminium **14a**

(a) Time 0 minutes

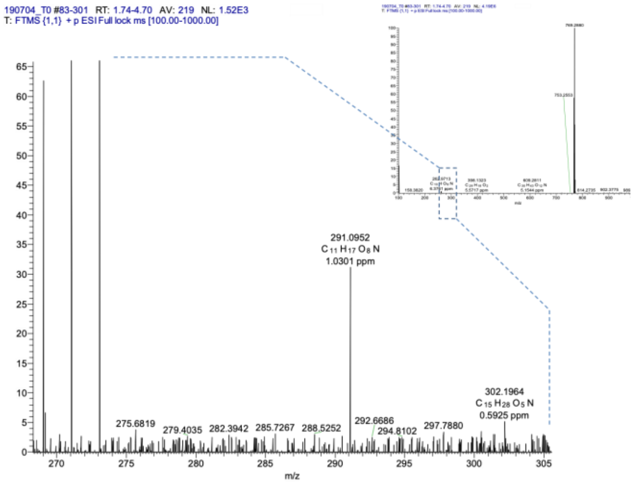

(b) Time 5 minutes

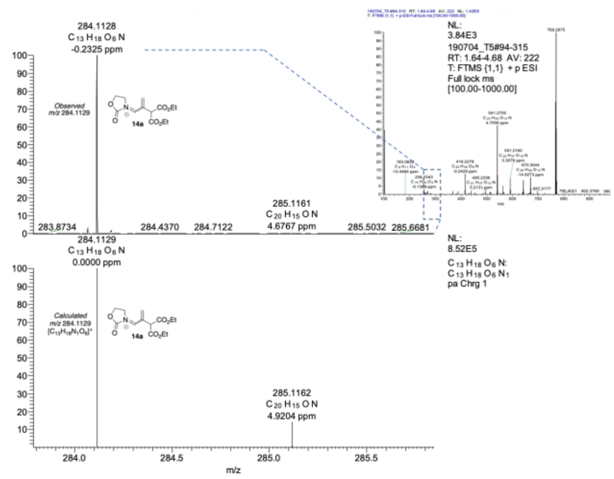

(c) Time 15 minutes

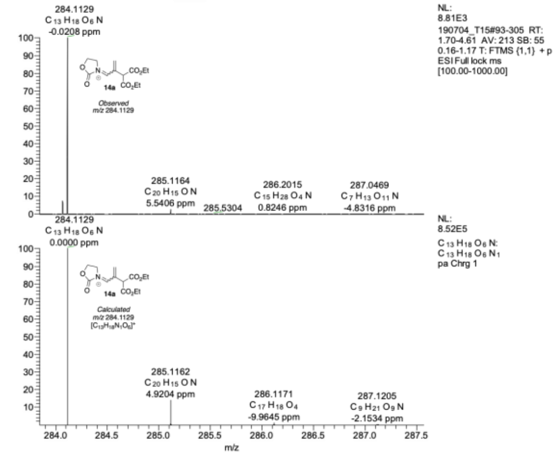

(d) Time 30 minutes

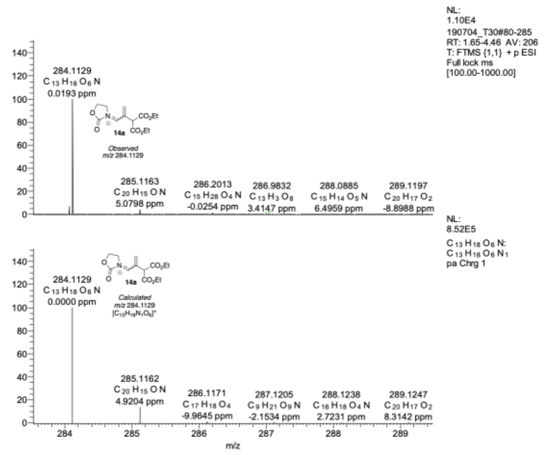

(e) Time 60 minutes

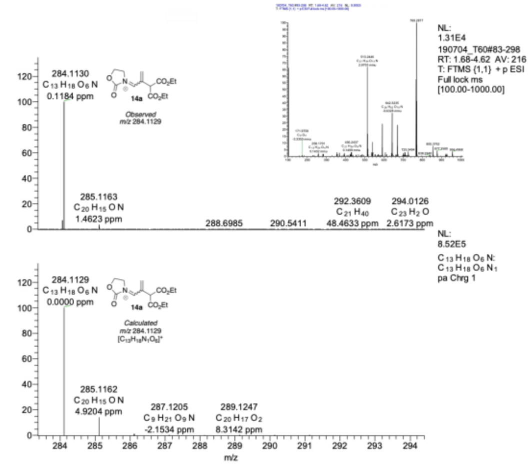

(f) Time 120 minutes

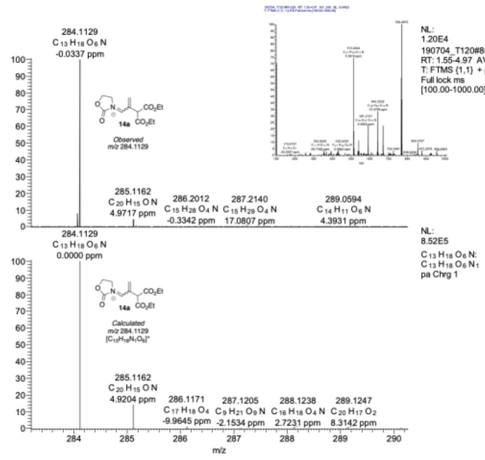

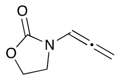

15

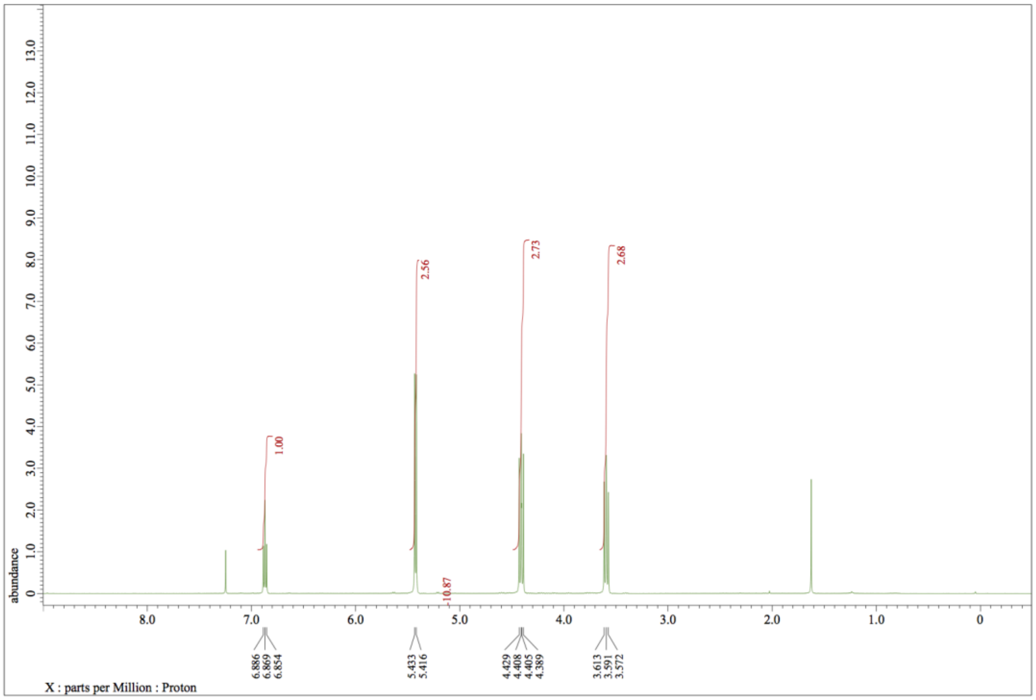

abundance
X : parts per Million : Proton
1.00
2.56
2.73
2.68
10.87
6.886
6.869
6.854
5.433
5.416
4.429
4.408
4.405
4.389
3.613
3.591
3.572

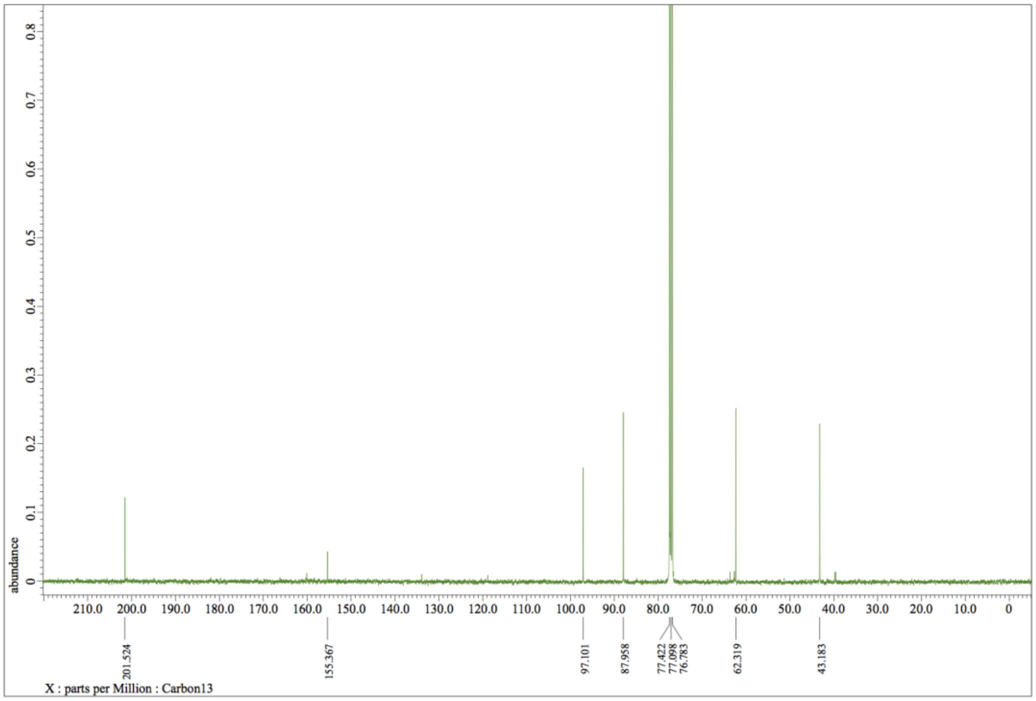

abundance
X : parts per Million : Carbon13
201.524
155.367
97.101
87.958
77.422
77.098
76.783
62.319
43.183

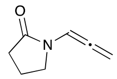

21

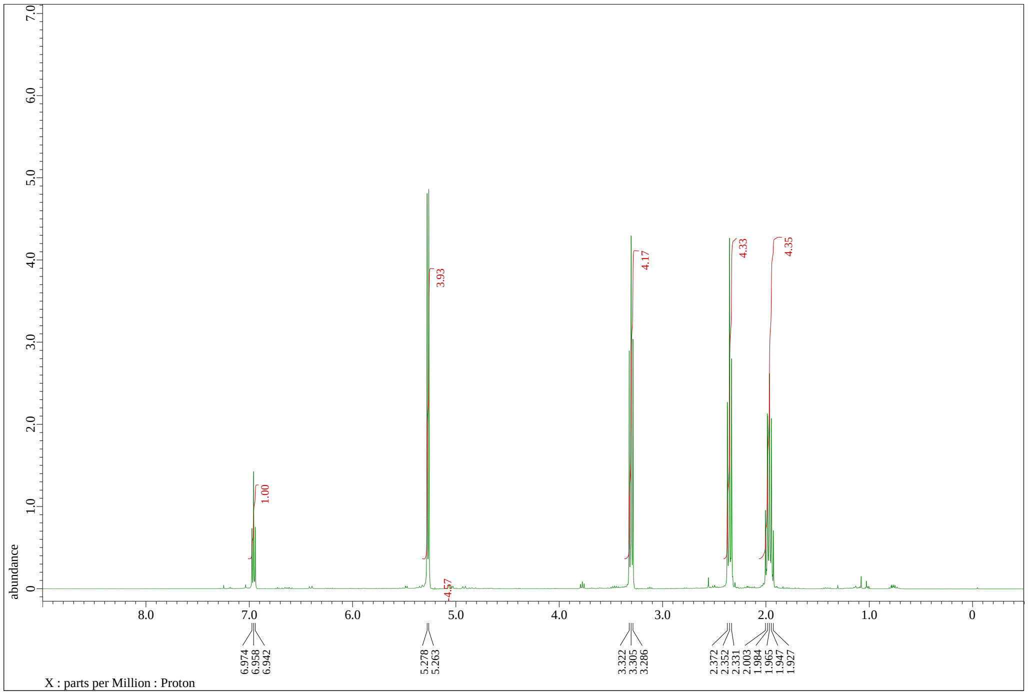

abundance
1.00
3.93
4.57
4.17
4.33
4.35
6.974
6.958
6.942
5.278
5.263
3.322
3.305
3.286
2.372
2.352
2.331
2.003
1.984
1.965
1.947
1.927
X : parts per Million : Proton

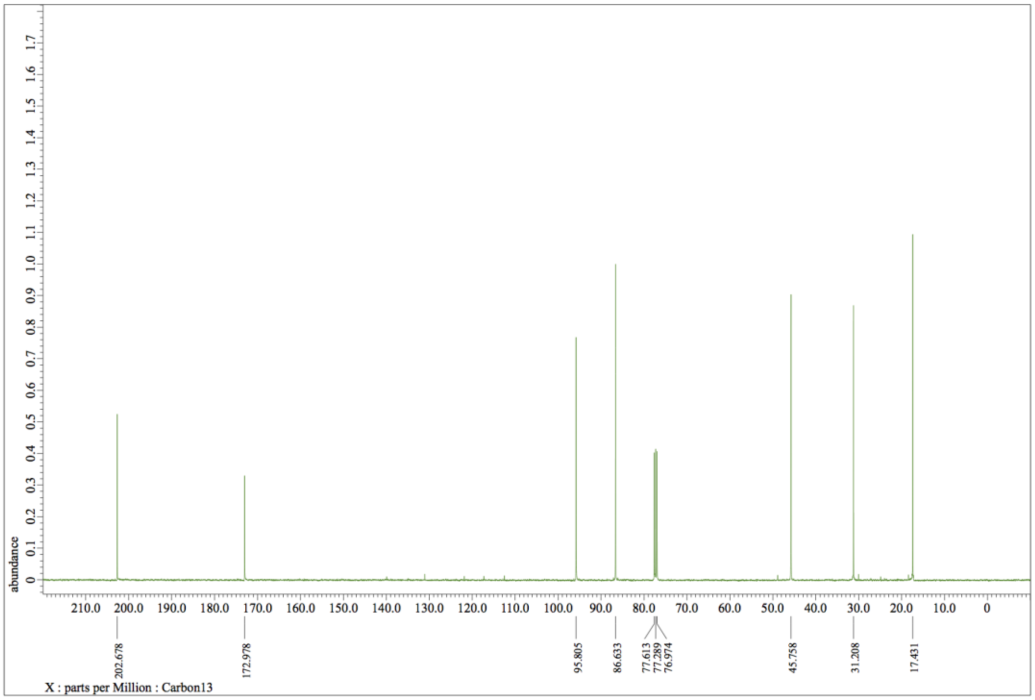

abundance
202.678
172.978
95.805
86.633
77.613
77.289
76.974
45.758
31.208
17.431
X : parts per Million : Carbon13

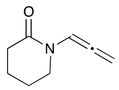

22

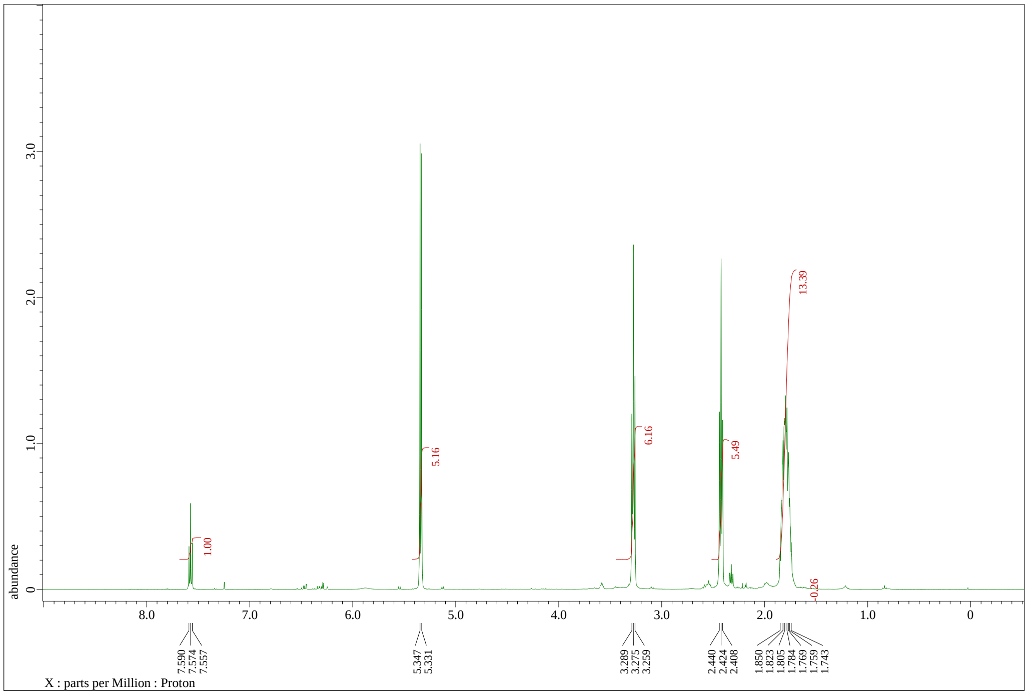
abundance
3.0
2.0
1.0
0
8.0
7.0
6.0
5.0
4.0
3.0
2.0
1.0
0
1.00
5.16
6.16
5.49
13.39
0.26
7.590
7.574
7.557
5.347
5.331
3.289
3.275
3.259
2.440
2.424
2.408
1.850
1.823
1.805
1.784
1.769
1.759
1.743
X : parts per Million : Proton

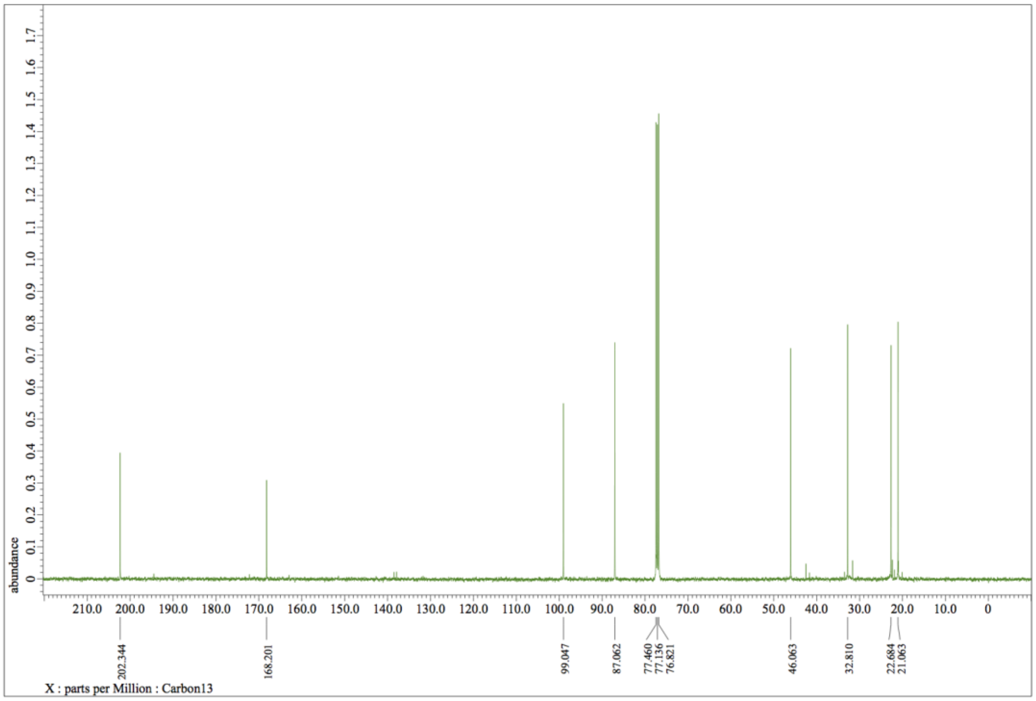
abundance
210.0
200.0
190.0
180.0
170.0
160.0
150.0
140.0
130.0
120.0
110.0
100.0
90.0
80.0
70.0
60.0
50.0
40.0
30.0
20.0
10.0
0
202.344
168.201
99.047
87.062
77.460
77.136
76.821
46.063
32.810
22.684
21.063
X : parts per Million : Carbon13

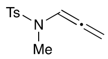

23

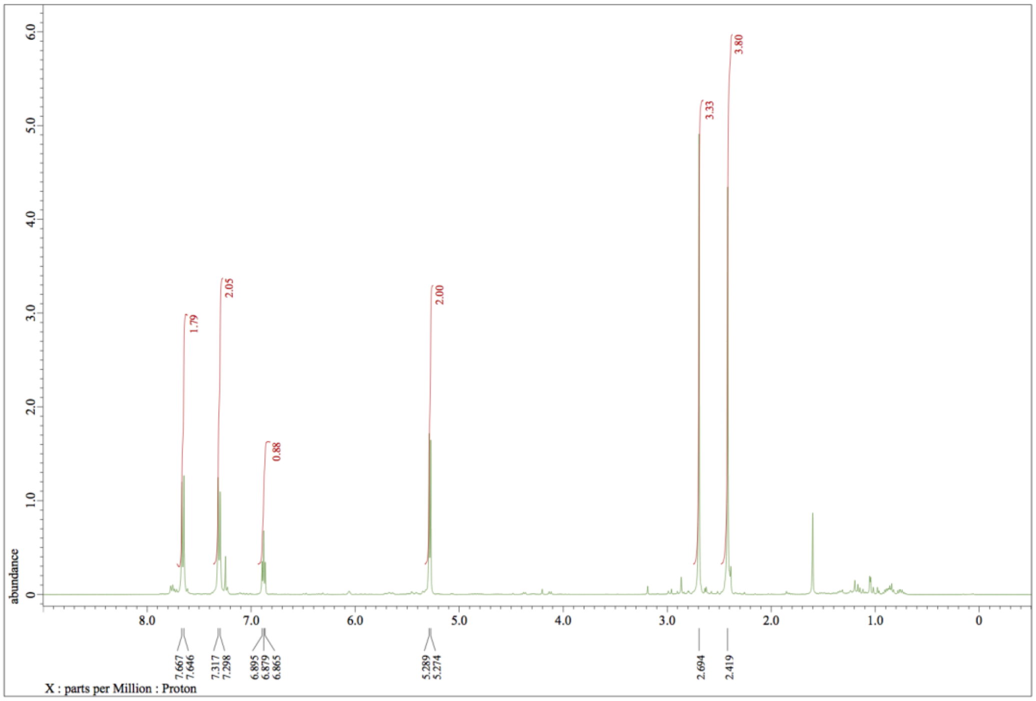
6.0
5.0
4.0
3.0
2.0
1.0
0
abundance
1.79
2.05
0.88
2.00
3.33
3.80
8.0
7.0
6.0
5.0
4.0
3.0
2.0
1.0
0
7.667
7.646
7.317
7.298
6.895
6.879
6.865
5.289
5.274
2.694
2.419
X : parts per Million : Proton

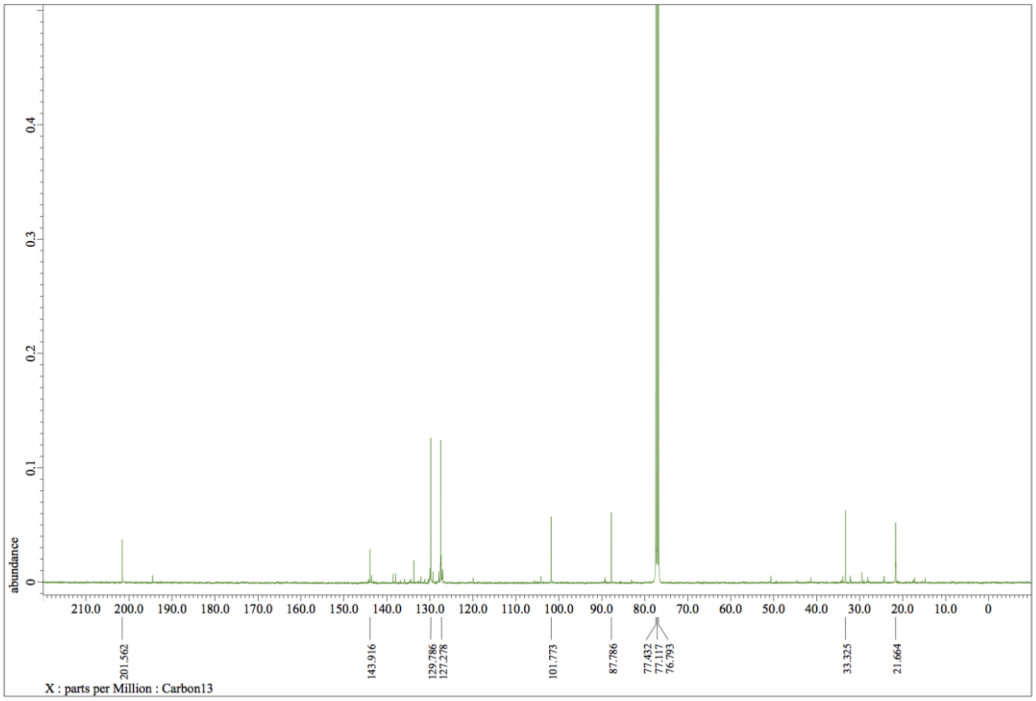
0.4
0.3
0.2
0.1
0
abundance
210.0
200.0
190.0
180.0
170.0
160.0
150.0
140.0
130.0
120.0
110.0
100.0
90.0
80.0
70.0
60.0
50.0
40.0
30.0
20.0
10.0
0
201.562
143.916
129.786
127.278
101.773
87.786
77.432
77.117
76.793
33.325
21.664
X : parts per Million : Carbon13

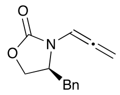

24

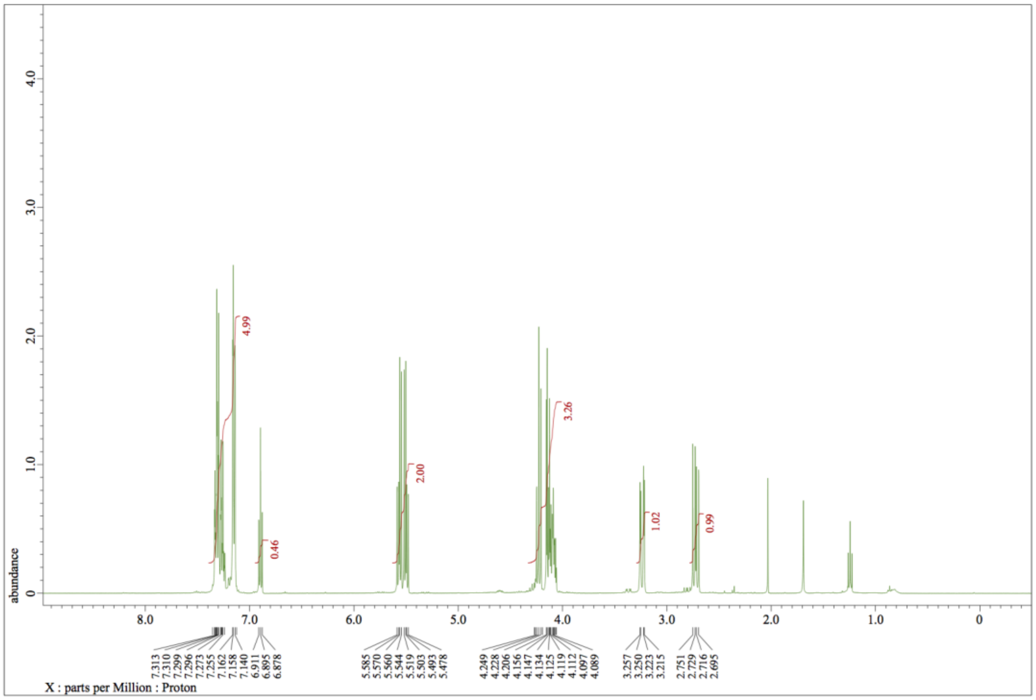
abundance
X : parts per Million : Proton
4.99
2.00
3.26
1.02
0.99
0.46
7.313
7.310
7.299
7.296
7.273
7.255
7.162
7.158
7.140
6.911
6.895
6.878
5.585
5.570
5.560
5.544
5.519
5.503
5.493
5.478
4.249
4.228
4.206
4.156
4.147
4.134
4.125
4.119
4.112
4.097
4.089
3.257
3.250
3.223
3.215
2.751
2.729
2.716
2.695

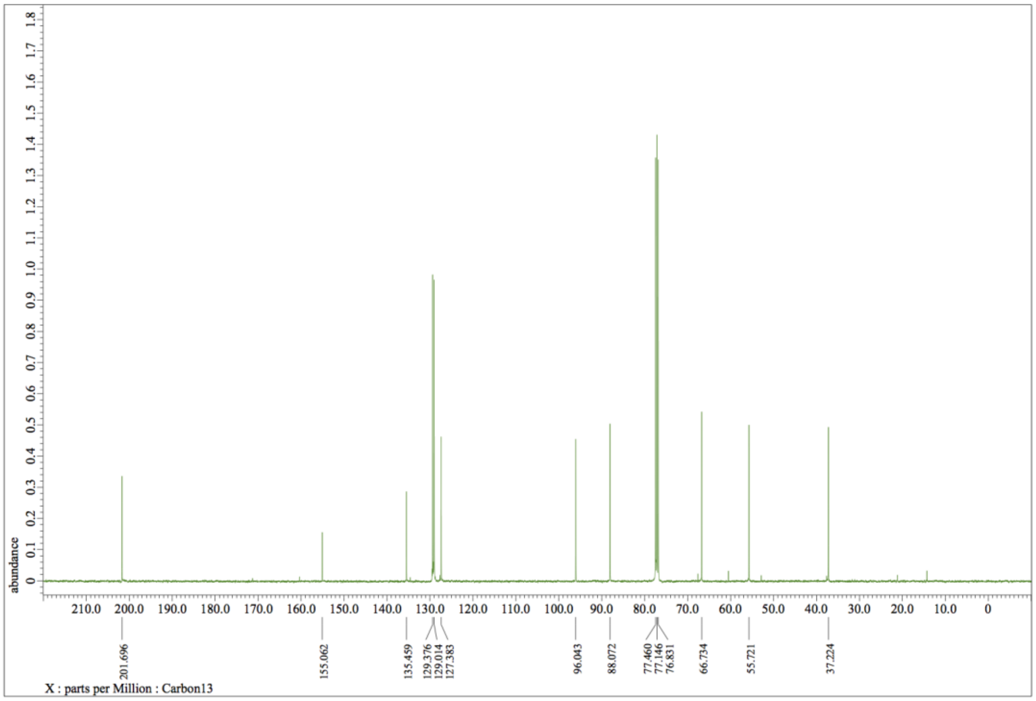
abundance
X : parts per Million : Carbon13
201.696
155.062
135.459
129.376
129.014
127.383
96.043
88.072
77.460
77.146
76.831
66.734
55.721
37.224

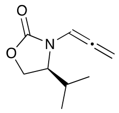

25

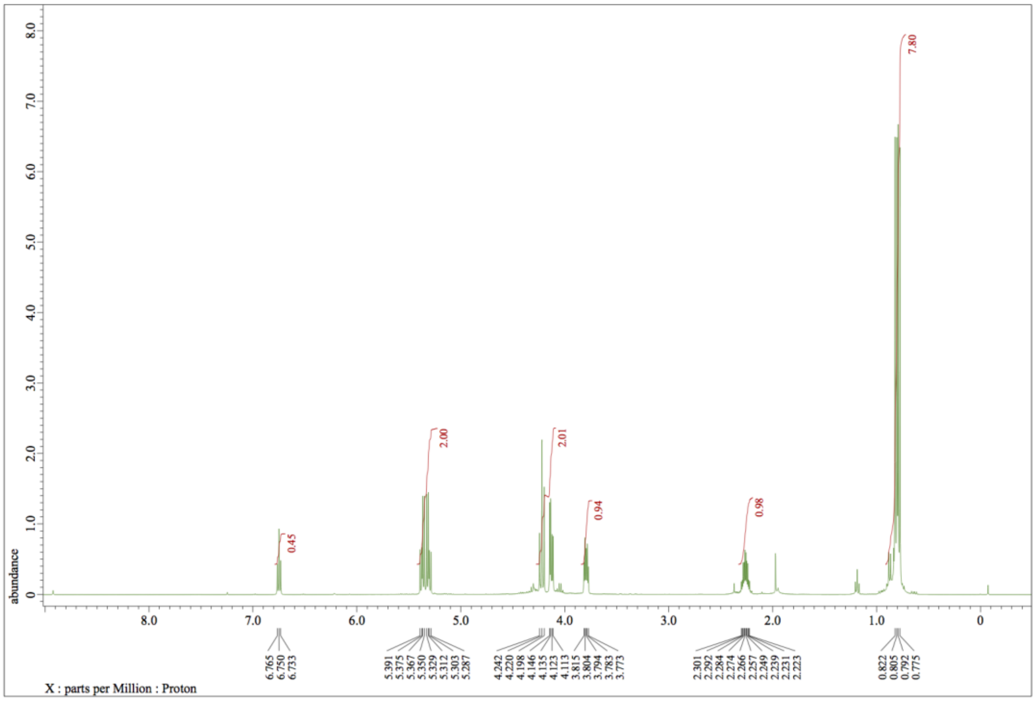
abundance
X : parts per Million : Proton
0.45
2.00
2.01
0.94
0.98
7.80
6.765
6.750
6.733
5.391
5.375
5.367
5.350
5.329
5.312
5.303
5.287
4.242
4.220
4.198
4.146
4.135
4.123
4.113
3.815
3.804
3.794
3.783
3.773
2.301
2.292
2.284
2.274
2.266
2.257
2.249
2.239
2.231
2.223
0.822
0.805
0.792
0.775

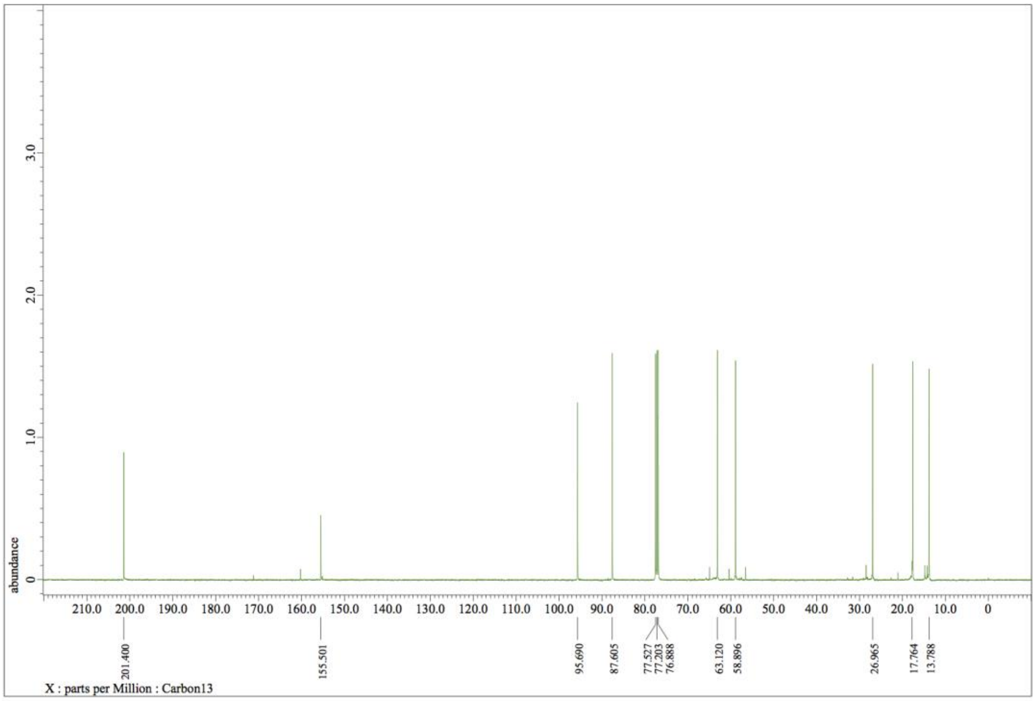
abundance
X : parts per Million : Carbon13
201.400
155.501
95.690
87.605
77.527
77.203
76.888
63.120
58.896
26.965
17.764
13.788

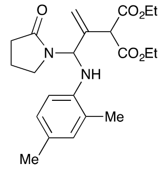
O
N
CO2Et
CO2Et
NH
Me
Me

26

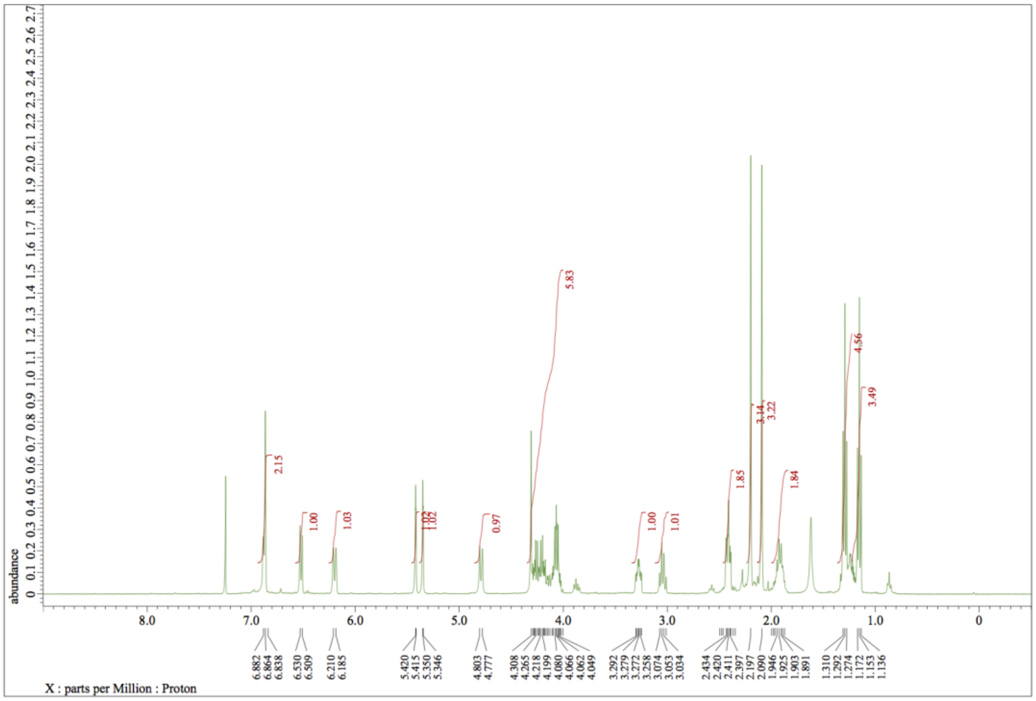
abundance
X : parts per Million : Proton

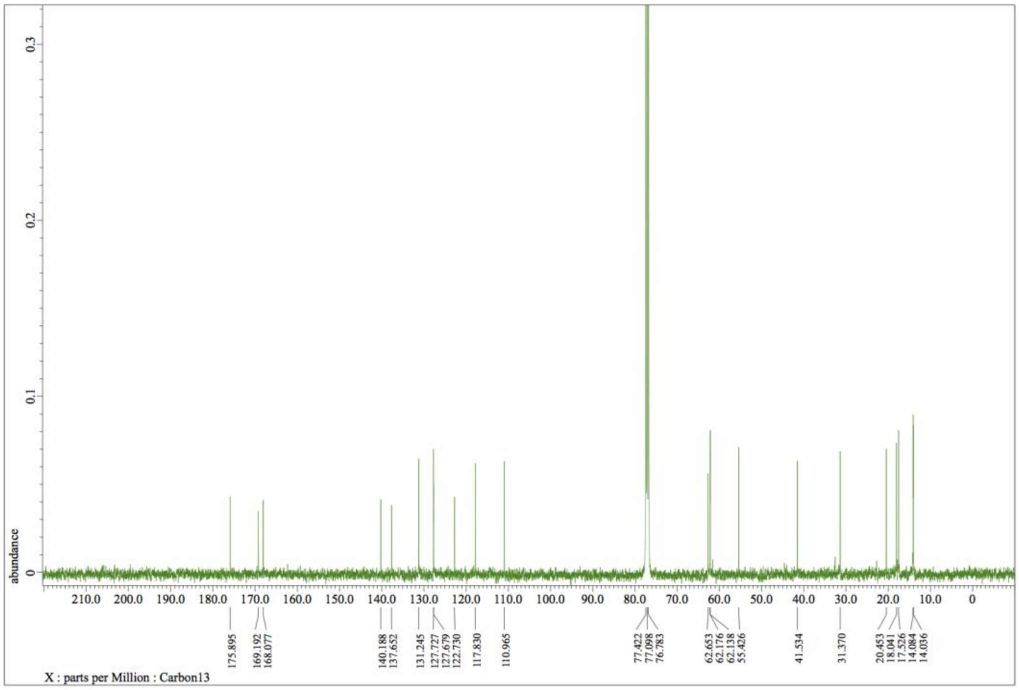
abundance
X : parts per Million : Carbon13

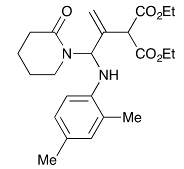

O
N
NH
CO2Et
CO2Et
Me
Me

27

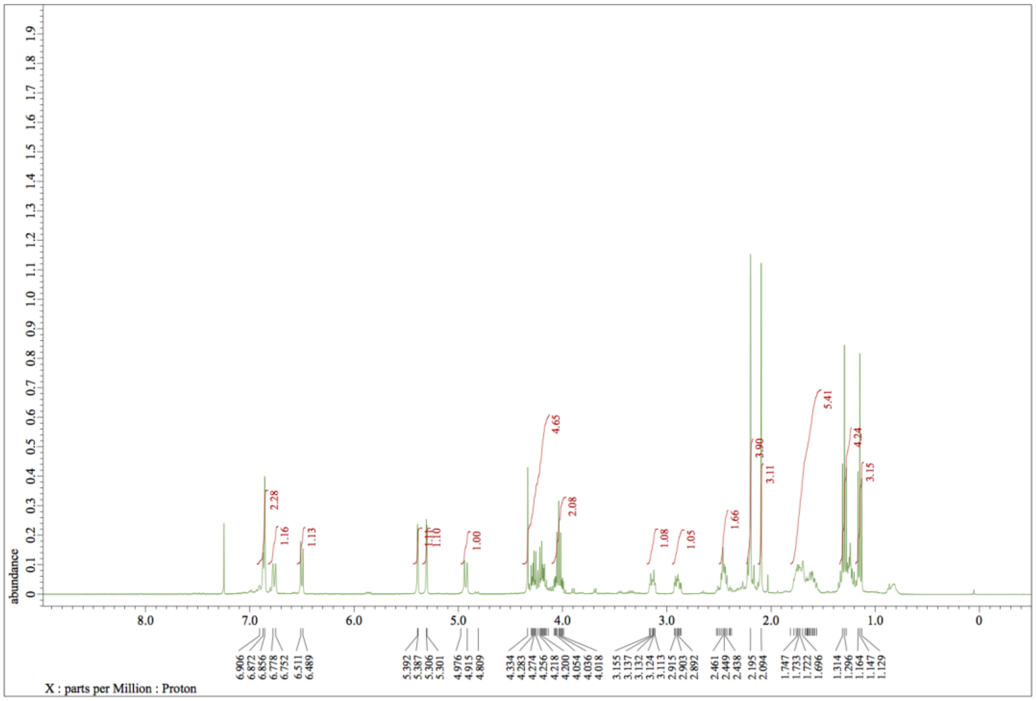

abundance
X : parts per Million : Proton
2.28
1.16
1.13
1.11
1.10
1.00
4.65
2.08
1.08
1.05
1.66
3.90
3.11
5.41
4.24
3.15
6.906
6.872
6.856
6.778
6.752
6.511
6.489
5.392
5.387
5.306
5.301
4.976
4.915
4.809
4.334
4.283
4.274
4.256
4.218
4.200
4.054
4.036
4.018
3.155
3.137
3.132
3.124
3.113
2.915
2.903
2.892
2.461
2.449
2.438
2.195
2.094
1.747
1.733
1.722
1.696
1.314
1.296
1.164
1.147
1.129

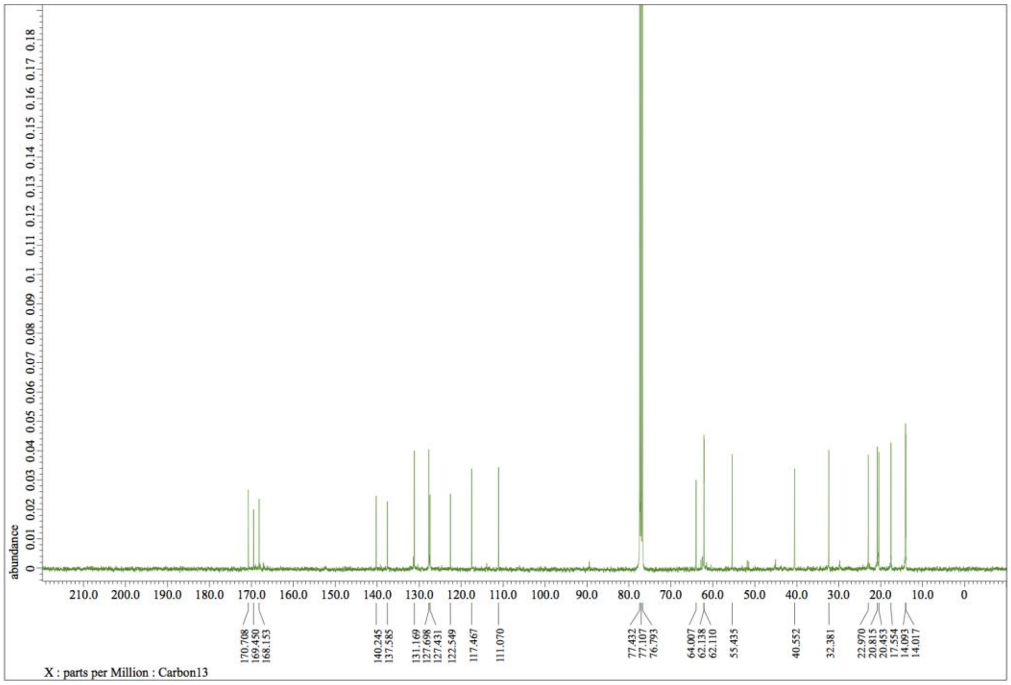

abundance
X : parts per Million : Carbon13
170.708
169.450
168.153
140.245
137.585
131.169
127.698
127.431
122.549
117.467
111.070
77.432
77.107
76.793
64.007
62.138
62.110
55.435
40.552
32.381
22.970
20.815
20.453
17.554
14.093
14.017

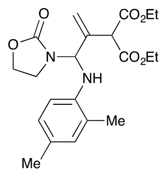
O
O
N
CO2Et
CO2Et
NH
Me
Me

28

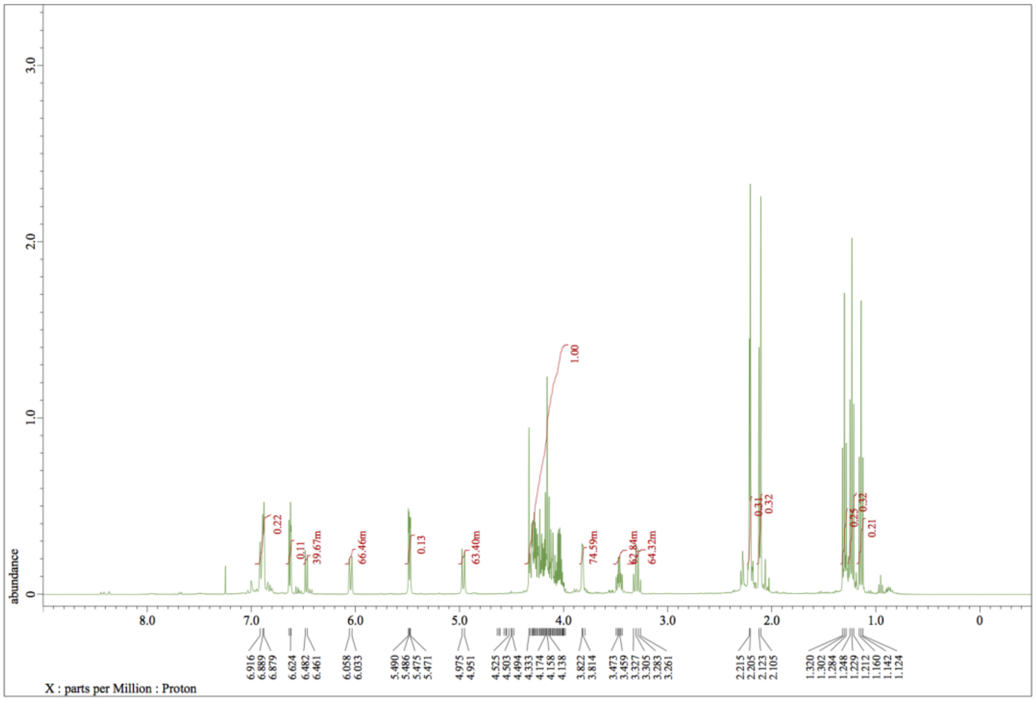
abundance
X : parts per Million : Proton
6.916
6.889
6.879
6.624
6.482
6.461
6.058
6.033
5.490
5.486
5.475
5.471
4.975
4.951
4.525
4.503
4.494
4.333
4.174
4.158
4.138
3.822
3.814
3.473
3.459
3.327
3.305
3.283
3.261
2.215
2.205
2.123
2.105
1.320
1.302
1.284
1.248
1.229
1.212
1.160
1.142
1.124

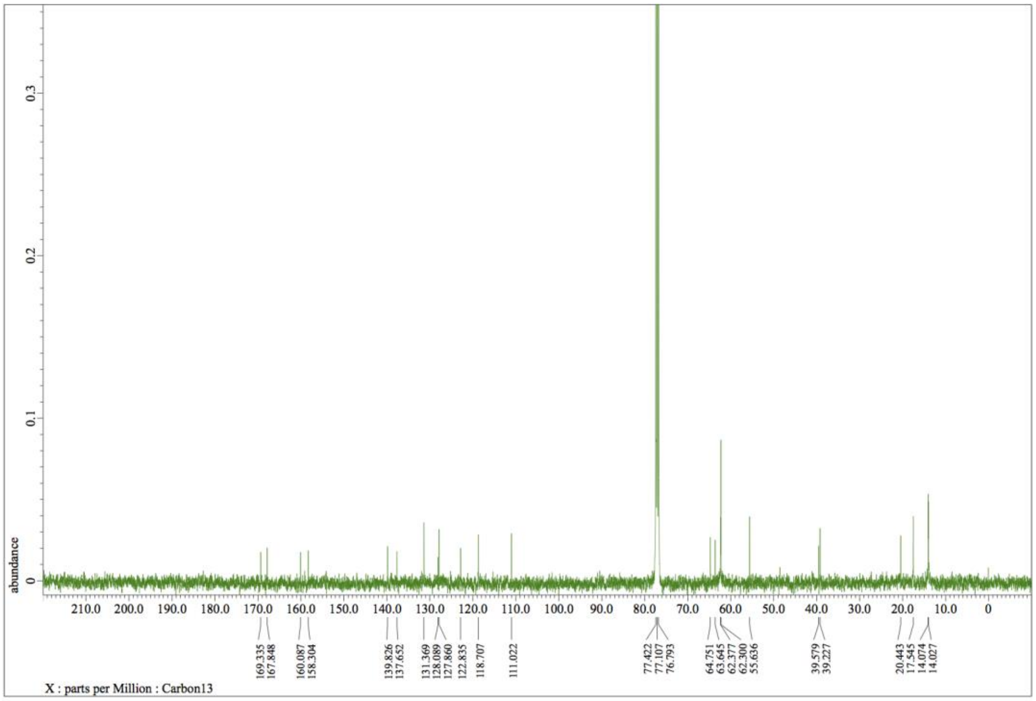
abundance
X : parts per Million : Carbon13
169.335
167.848
160.087
158.304
139.826
137.652
131.369
128.089
127.860
122.835
118.707
111.022
77.422
77.107
76.793
64.751
63.645
62.377
62.300
55.636
39.579
39.227
20.443
17.545
14.074
14.027

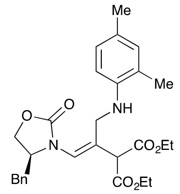

Z-**30**

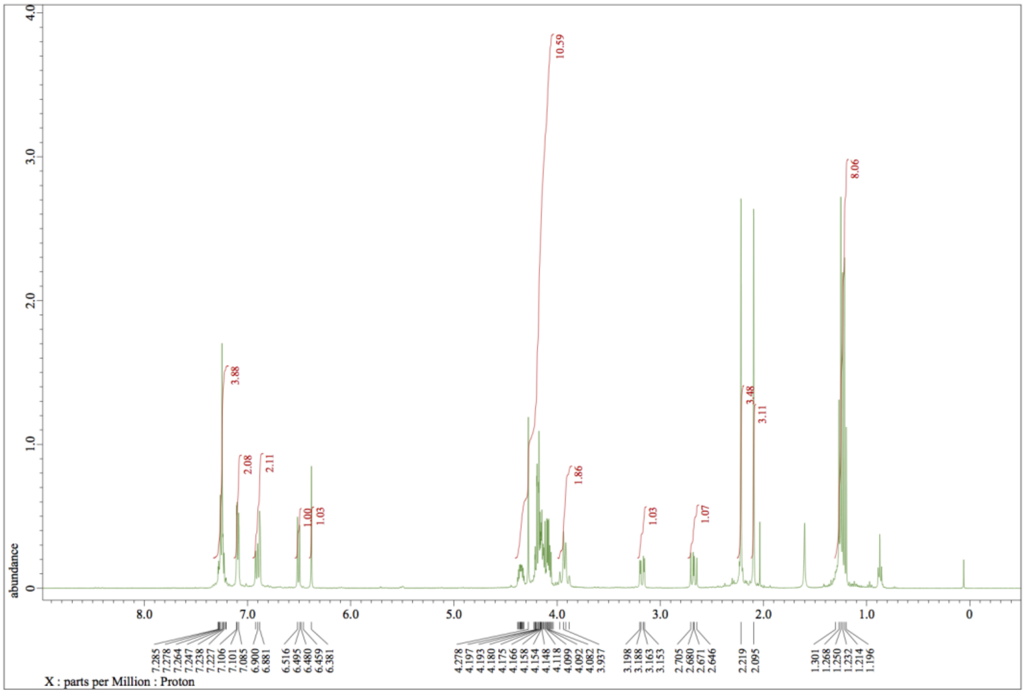

X : parts per Million : Proton
abundance

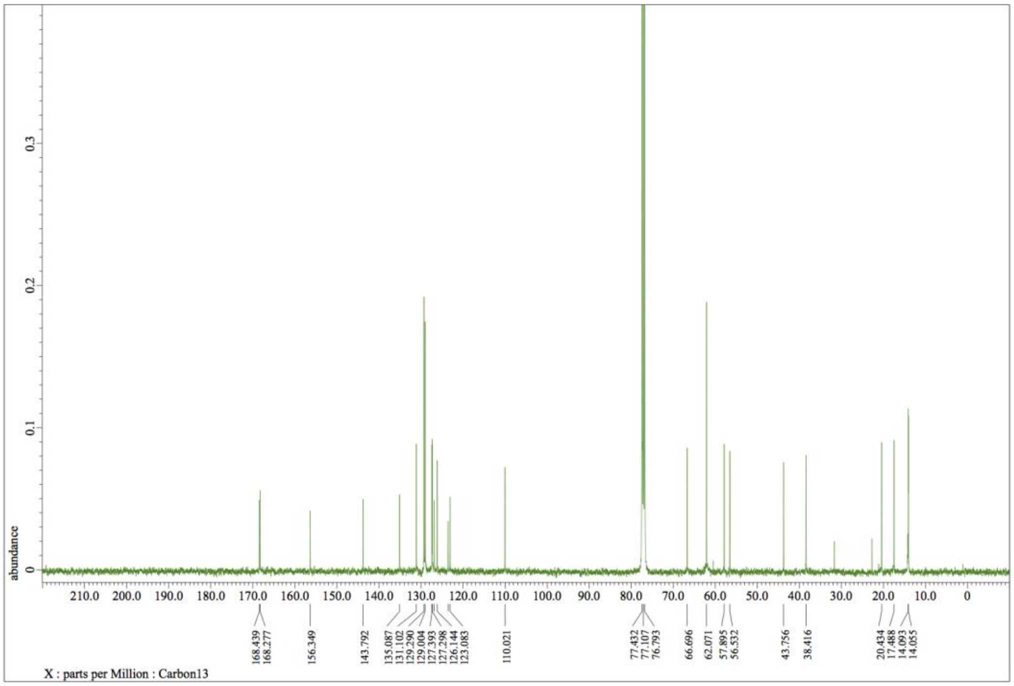

X : parts per Million : Carbon13
abundance

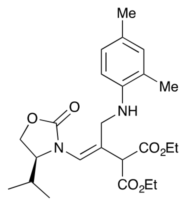
Me
Me
NH
O
O
N
CO2Et
CO2Et

Z-**31**

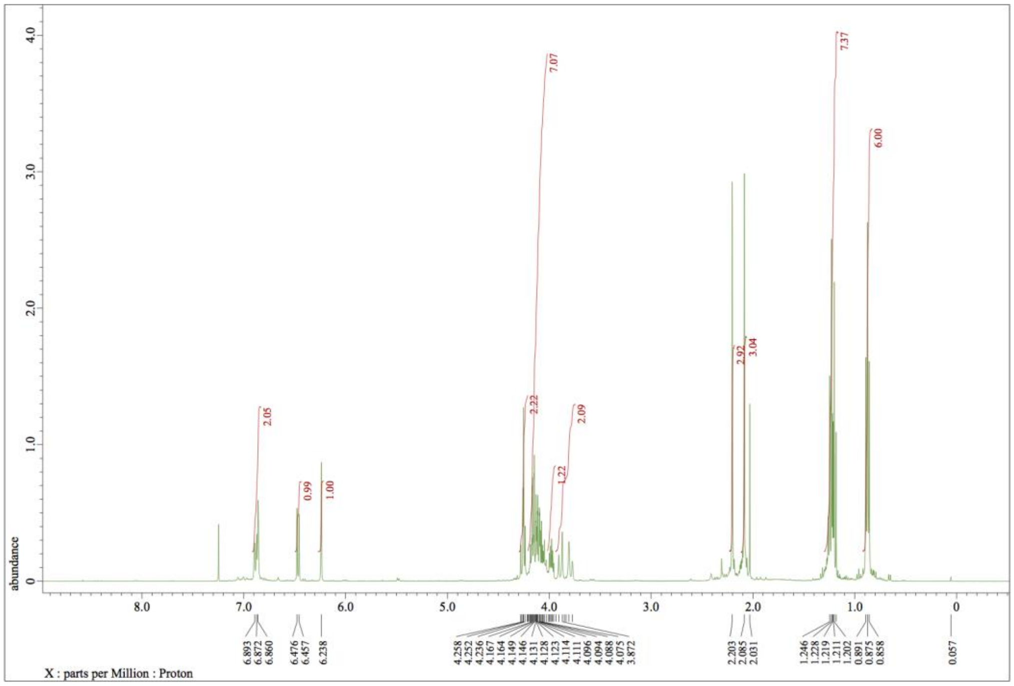
abundance
X : parts per Million : Proton
2.05
0.99
1.00
2.22
7.07
1.22
2.09
2.92
3.04
7.37
6.00
6.893
6.872
6.860
6.476
6.457
6.238
4.258
4.252
4.236
4.167
4.164
4.149
4.146
4.131
4.128
4.123
4.114
4.111
4.096
4.094
4.088
4.075
3.872
2.203
2.085
2.031
1.246
1.228
1.219
1.211
1.202
0.891
0.875
0.858
0.057

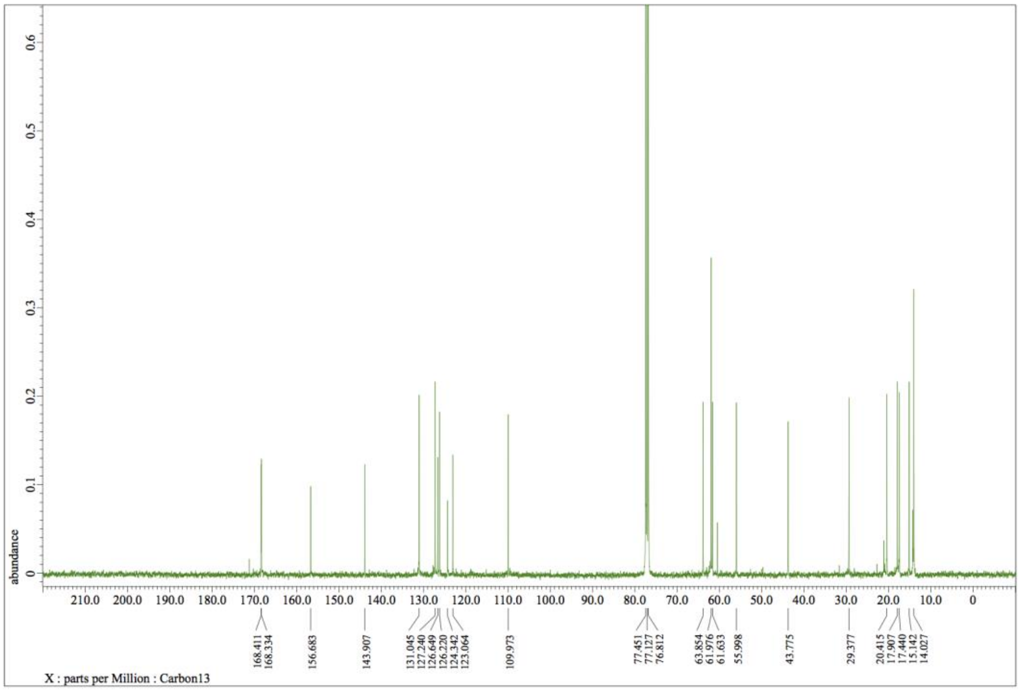
abundance
X : parts per Million : Carbon13
168.411
168.334
156.683
143.907
131.045
127.240
126.649
126.220
124.342
123.064
109.973
77.451
77.127
76.812
63.854
61.976
61.633
55.998
43.775
29.377
20.415
17.907
17.440
15.142
14.027

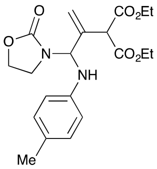
O
O
N
CO2Et
CO2Et
NH
Me

32

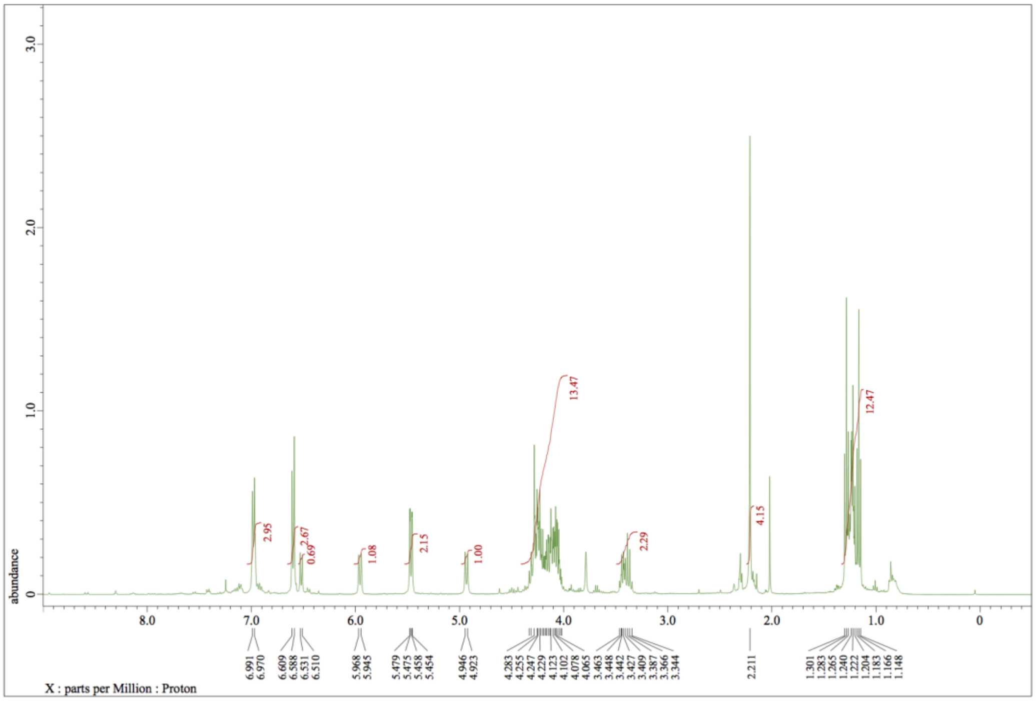
abundance
X : parts per Million : Proton
13.47
12.47
4.15
2.95
2.67
0.69
1.08
2.15
1.00
2.29
6.991
6.970
6.609
6.588
6.531
6.510
5.968
5.945
5.479
5.475
5.458
5.454
4.946
4.923
4.283
4.255
4.247
4.229
4.123
4.102
4.078
4.065
3.463
3.448
3.442
3.427
3.409
3.387
3.366
3.344
2.211
1.301
1.283
1.265
1.240
1.222
1.204
1.183
1.166
1.148

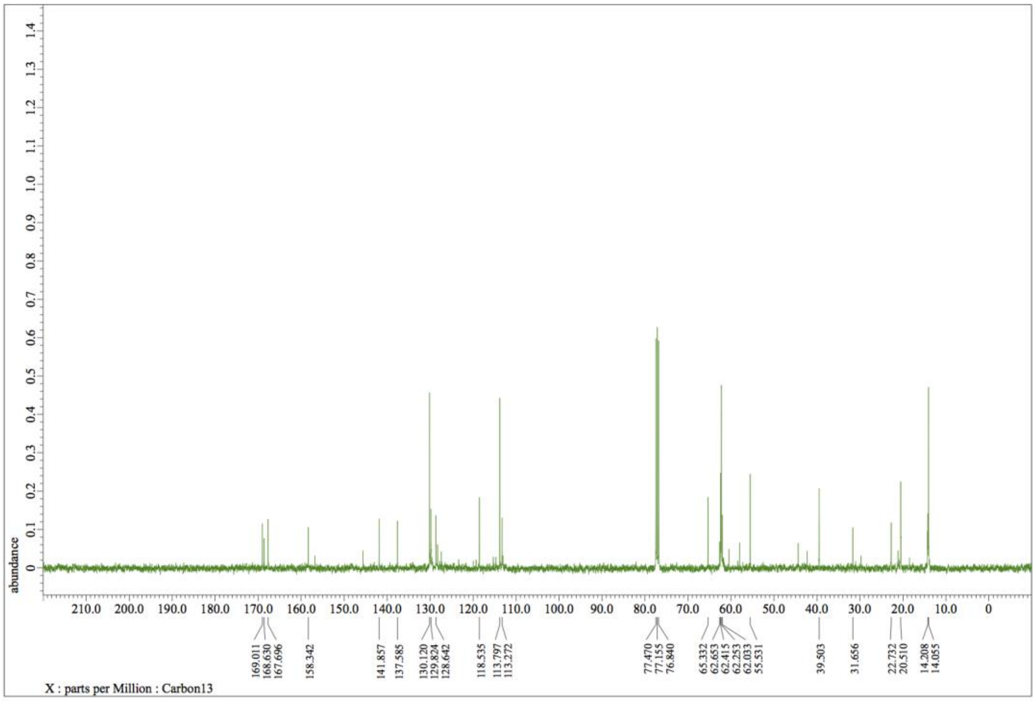
abundance
X : parts per Million : Carbon13
169.011
168.630
167.696
158.342
141.857
137.585
130.120
129.824
128.642
118.535
113.797
113.272
77.470
77.155
76.840
65.332
62.653
62.415
62.253
62.033
55.531
39.503
31.656
22.732
20.510
14.208
14.055

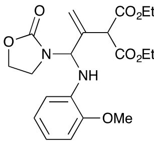
O
O
N
CO2Et
CO2Et
NH
OMe

33

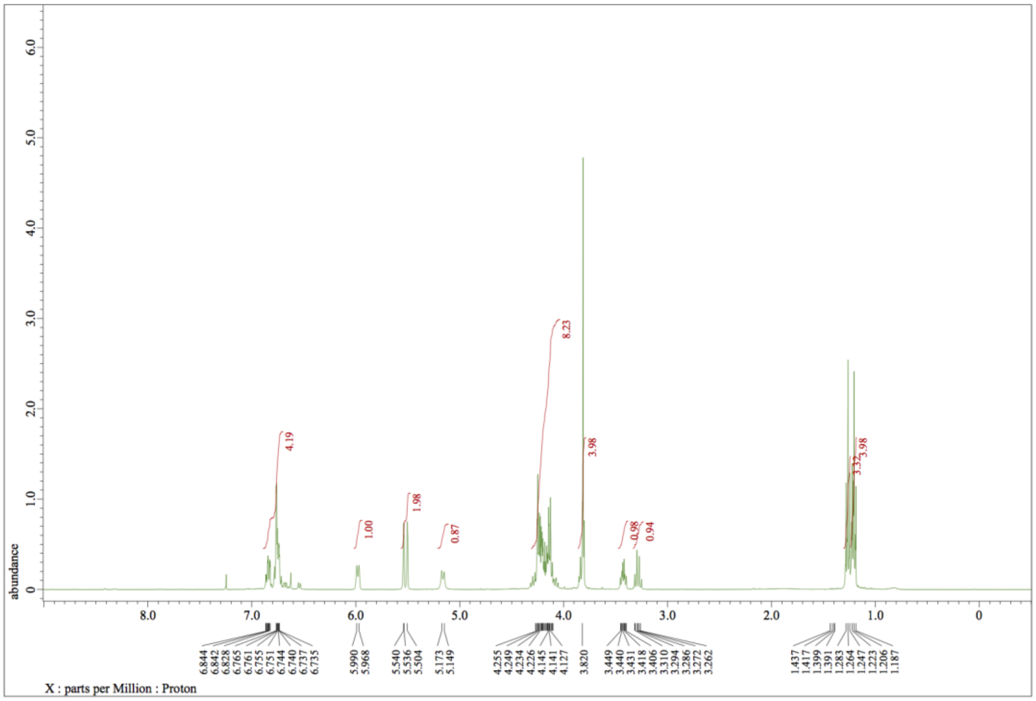
abundance
6.0
5.0
4.0
3.0
2.0
1.0
0
4.19
1.00
1.98
0.87
8.23
3.98
0.98
0.94
3.32
3.98
8.0
7.0
6.0
5.0
4.0
3.0
2.0
1.0
0
6.844 6.842 6.828 6.765 6.761 6.755 6.751 6.744 6.740 6.737 6.735
5.990 5.968
5.540 5.536 5.504
5.173 5.149
4.255 4.249 4.234 4.226 4.145 4.141 4.127
3.820
3.449 3.440 3.431 3.418 3.406 3.310 3.294 3.286 3.272 3.262
1.437 1.417 1.399 1.391 1.283 1.264 1.247 1.223 1.206 1.187
X : parts per Million : Proton

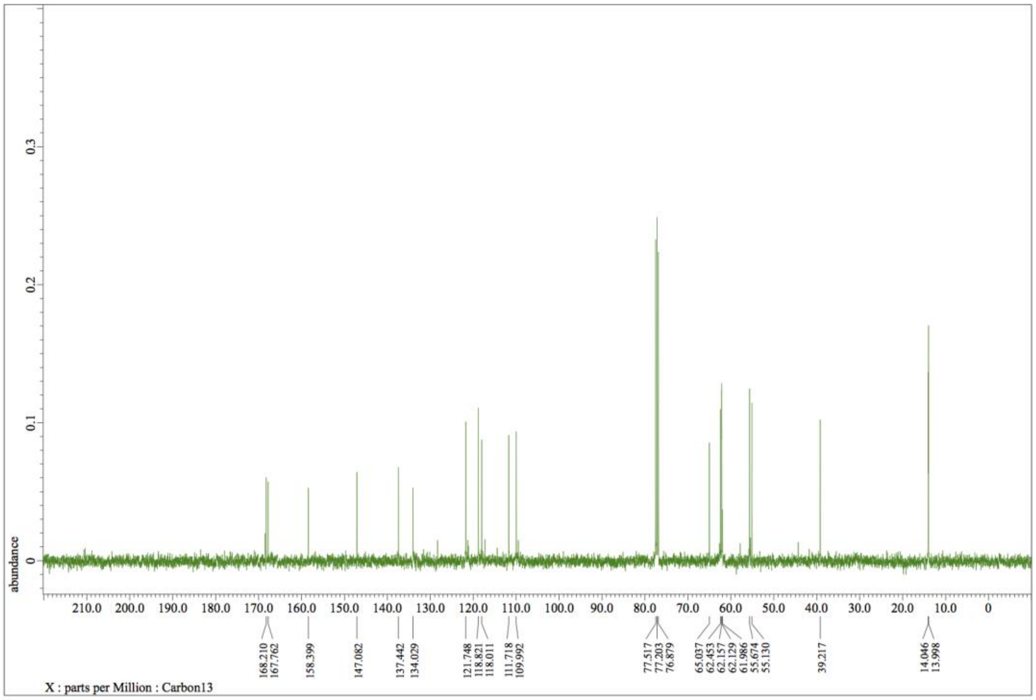
abundance
0.3
0.2
0.1
0
210.0
200.0
190.0
180.0
170.0
160.0
150.0
140.0
130.0
120.0
110.0
100.0
90.0
80.0
70.0
60.0
50.0
40.0
30.0
20.0
10.0
0
168.210 167.762
158.399
147.082
137.442 134.029
121.748 118.821 118.011
111.718 109.992
77.517 77.203 76.879
65.037 62.453 62.157 62.129 61.986 55.674 55.130
39.217
14.046 13.998
X : parts per Million : Carbon13

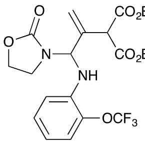

O
O
N
NH
CO2Et
CO2Et
OCF3

34

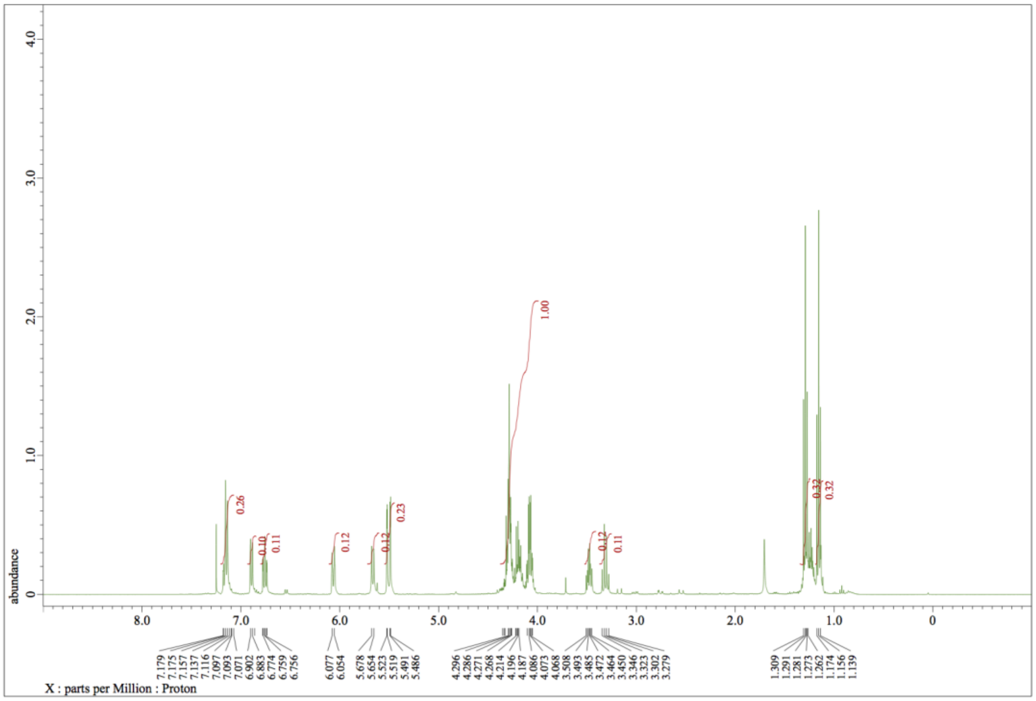

abundance
4.0
3.0
2.0
1.0
0
0.26
0.10
0.11
0.12
0.12
0.23
1.00
0.12
0.11
0.32
0.32
8.0
7.0
6.0
5.0
4.0
3.0
2.0
1.0
0
7.179 7.175 7.157 7.137 7.116 7.097 7.093 7.071 6.902 6.883 6.774 6.759 6.756
6.077 6.054
5.678 5.654 5.523 5.519 5.491 5.486
4.296 4.286 4.271 4.268 4.214 4.196 4.187 4.086 4.073 4.068 3.508 3.493 3.485 3.472 3.464 3.450 3.346 3.323 3.302 3.279
1.309 1.291 1.281 1.273 1.262 1.174 1.156 1.139
X : parts per Million : Proton

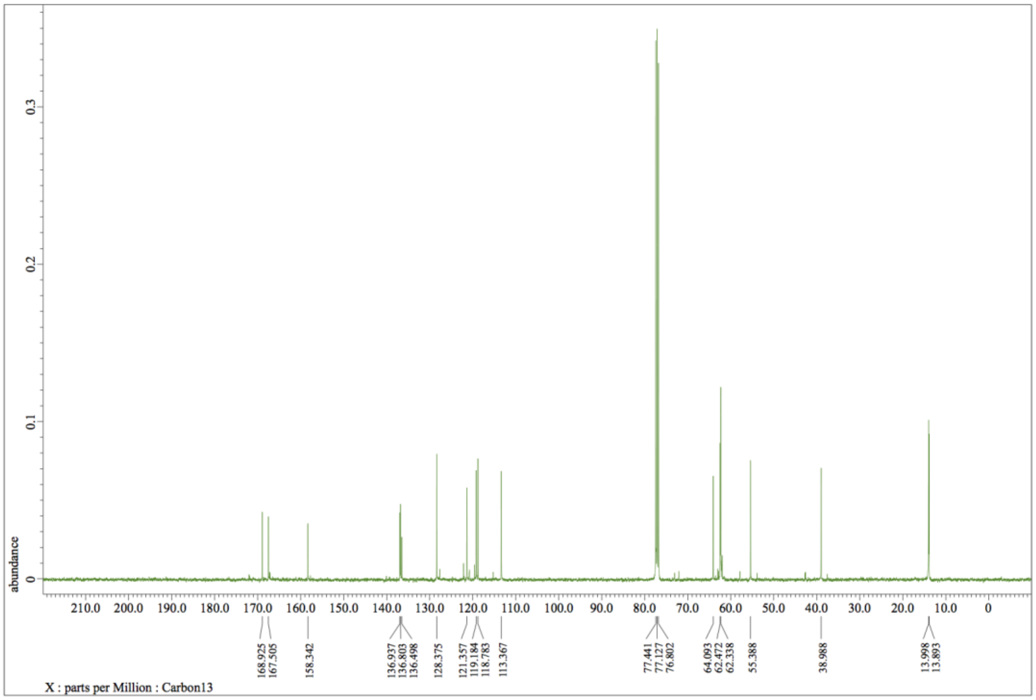

abundance
0.3
0.2
0.1
0
210.0 200.0 190.0 180.0 170.0 160.0 150.0 140.0 130.0 120.0 110.0 100.0 90.0 80.0 70.0 60.0 50.0 40.0 30.0 20.0 10.0 0
168.925 167.505
158.342
136.937 136.803 136.498
128.375
121.357 119.184 118.783
113.367
77.441 77.127 76.802
64.093 62.472 62.338
55.388
38.988
13.998 13.893
X : parts per Million : Carbon13

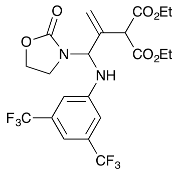
O
O
N
CO2Et
CO2Et
NH
F3C
CF3

35

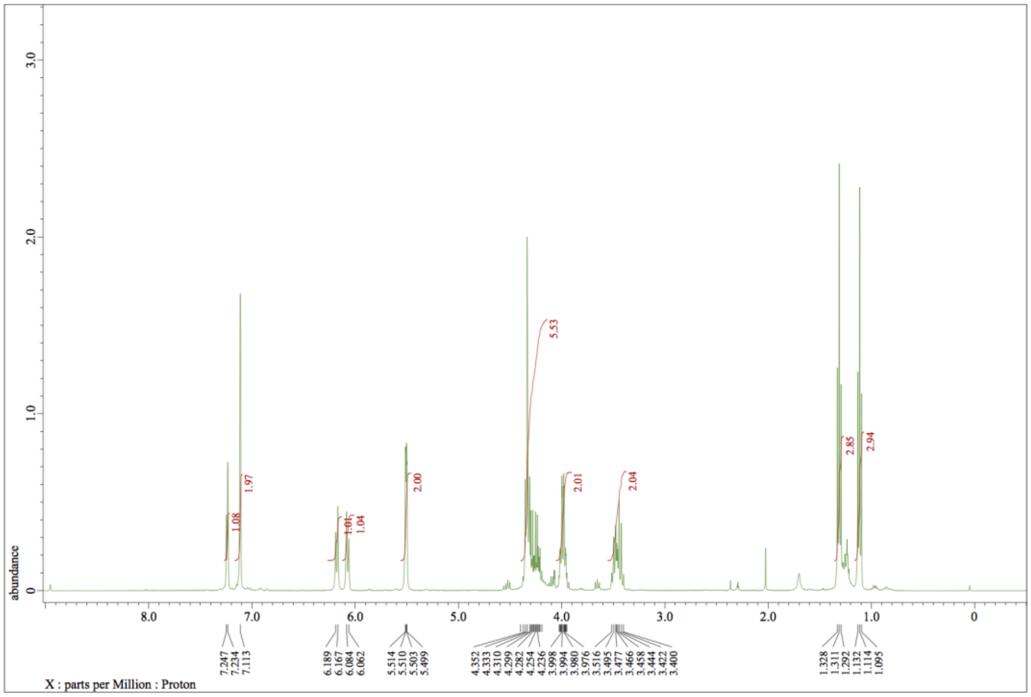
3.0
2.0
1.0
0
abundance
1.08
1.97
1.01
1.04
2.00
5.53
2.01
2.04
2.85
2.94
8.0
7.0
6.0
5.0
4.0
3.0
2.0
1.0
0
7.247
7.234
7.113
6.189
6.167
6.084
6.062
5.514
5.510
5.503
5.499
4.352
4.333
4.310
4.299
4.282
4.254
4.236
3.998
3.994
3.980
3.976
3.516
3.495
3.477
3.466
3.458
3.444
3.422
3.400
1.328
1.311
1.292
1.132
1.114
1.095
X : parts per Million : Proton

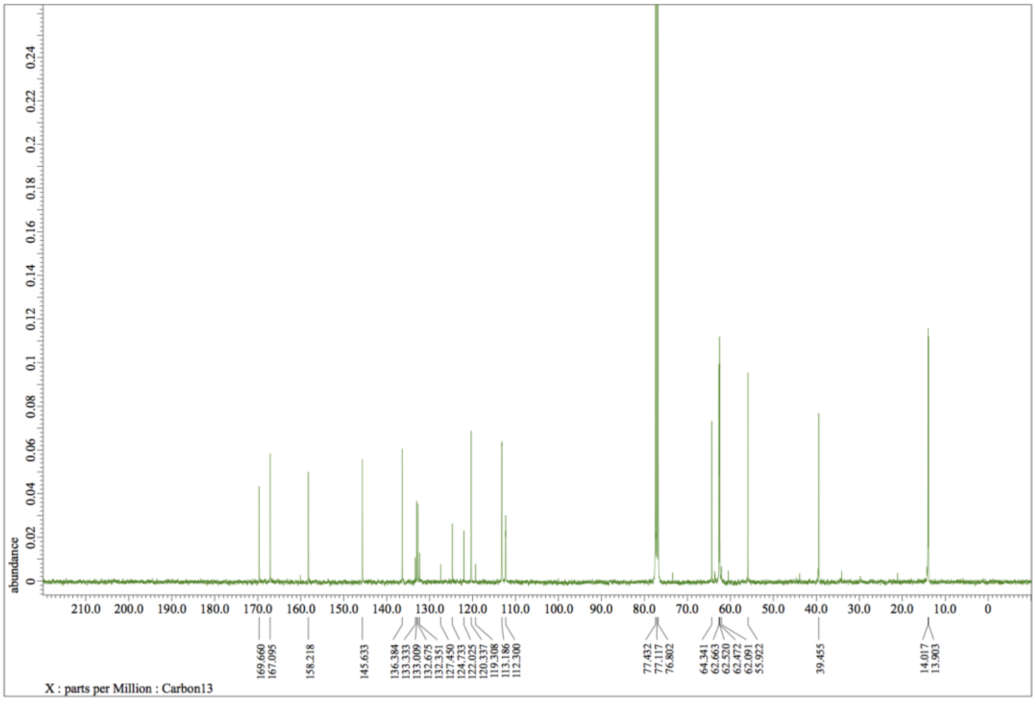
0.24
0.22
0.2
0.18
0.16
0.14
0.12
0.1
0.08
0.06
0.04
0.02
0
abundance
210.0
200.0
190.0
180.0
170.0
160.0
150.0
140.0
130.0
120.0
110.0
100.0
90.0
80.0
70.0
60.0
50.0
40.0
30.0
20.0
10.0
0
169.660
167.095
158.218
145.633
136.384
133.333
133.009
132.675
132.351
127.450
124.733
122.025
120.337
119.308
113.186
112.300
77.432
77.117
76.802
64.341
62.663
62.520
62.472
62.091
55.922
39.455
14.017
13.903
X : parts per Million : Carbon13

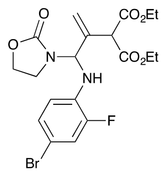

36

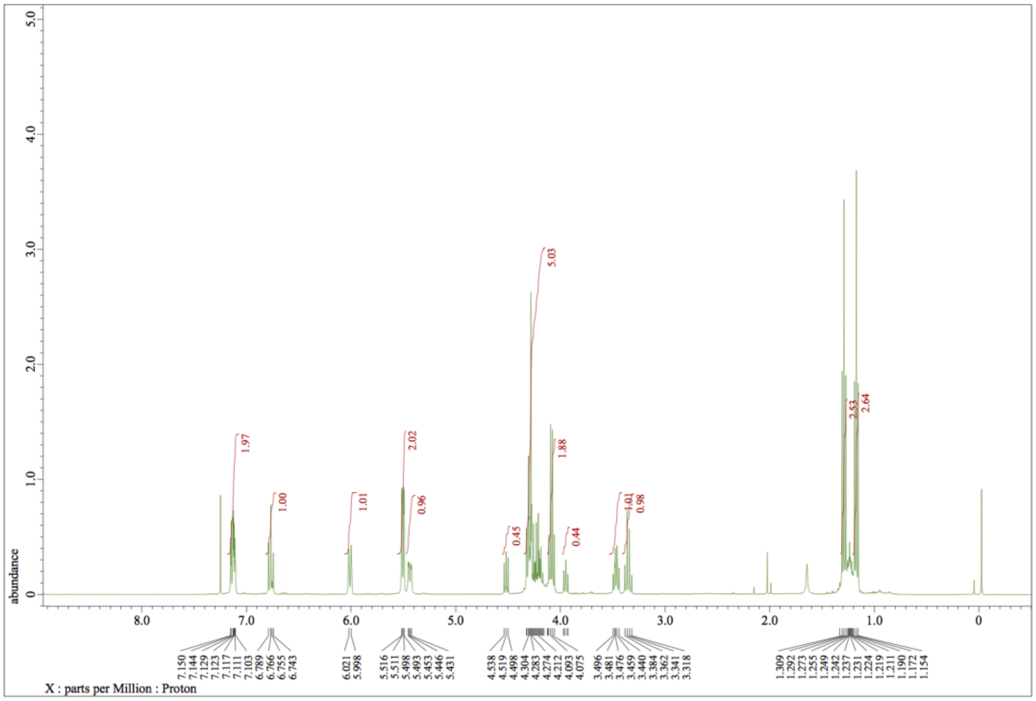
abundance
X : parts per Million : Proton

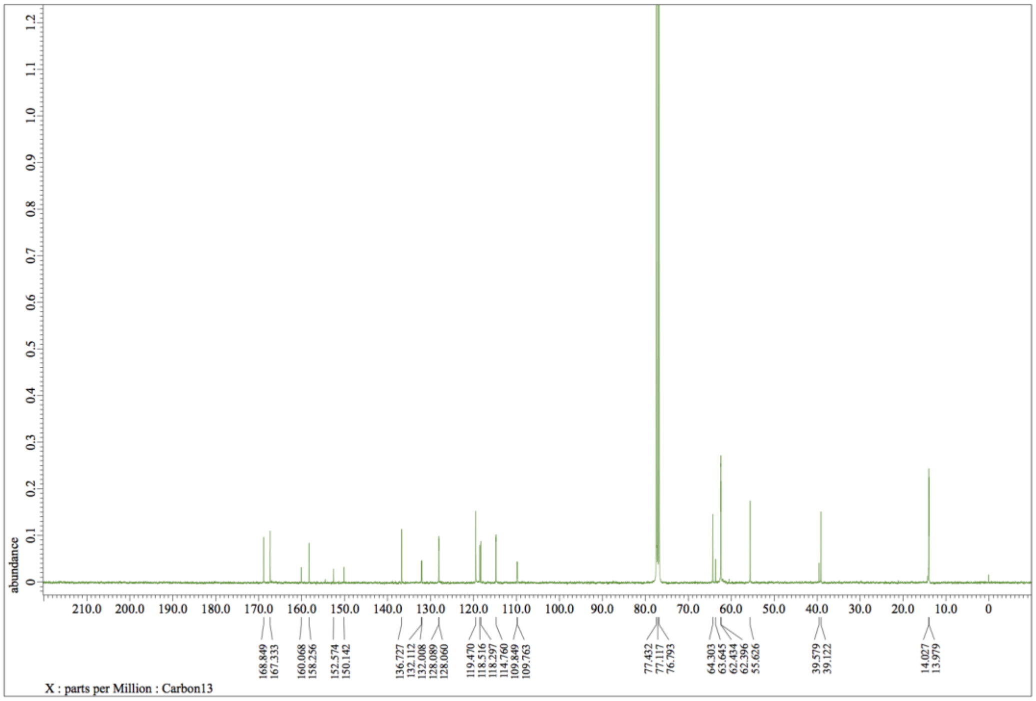
abundance
X : parts per Million : Carbon13

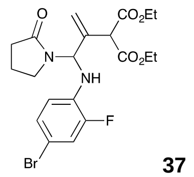
O
CO2Et
N
CO2Et
NH
F
Br

37

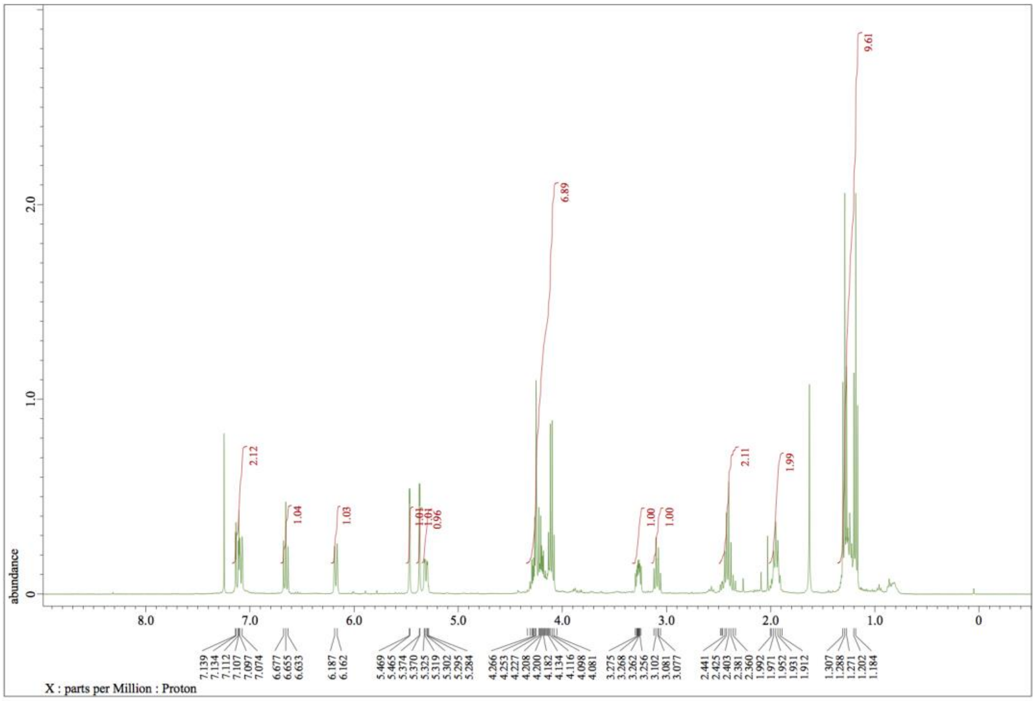
abundance
X : parts per Million : Proton
2.12
1.04
1.03
1.01
1.01
0.96
6.89
1.00
1.00
2.11
1.99
9.61
7.139
7.134
7.112
7.107
7.097
7.074
6.677
6.655
6.633
6.187
6.162
5.469
5.465
5.374
5.370
5.325
5.319
5.302
5.295
5.284
4.266
4.253
4.227
4.208
4.200
4.182
4.134
4.116
4.098
4.081
3.275
3.268
3.262
3.256
3.102
3.081
3.077
2.441
2.425
2.403
2.381
2.360
1.992
1.971
1.952
1.931
1.912
1.307
1.288
1.271
1.202
1.184

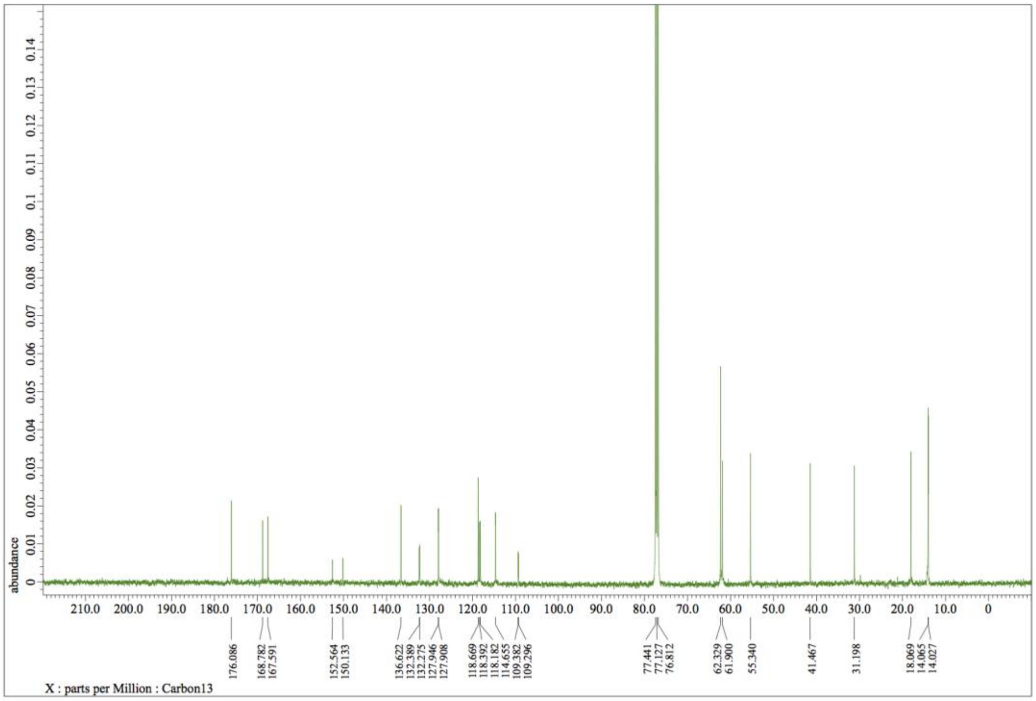
abundance
X : parts per Million : Carbon13
176.086
168.782
167.591
152.564
150.133
136.622
132.389
132.275
127.946
127.908
118.669
118.392
118.182
114.655
109.382
109.296
77.441
77.127
76.812
62.329
61.900
55.340
41.467
31.198
18.069
14.065
14.027

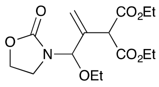
O
O
N
OEt
CO2Et
CO2Et

39

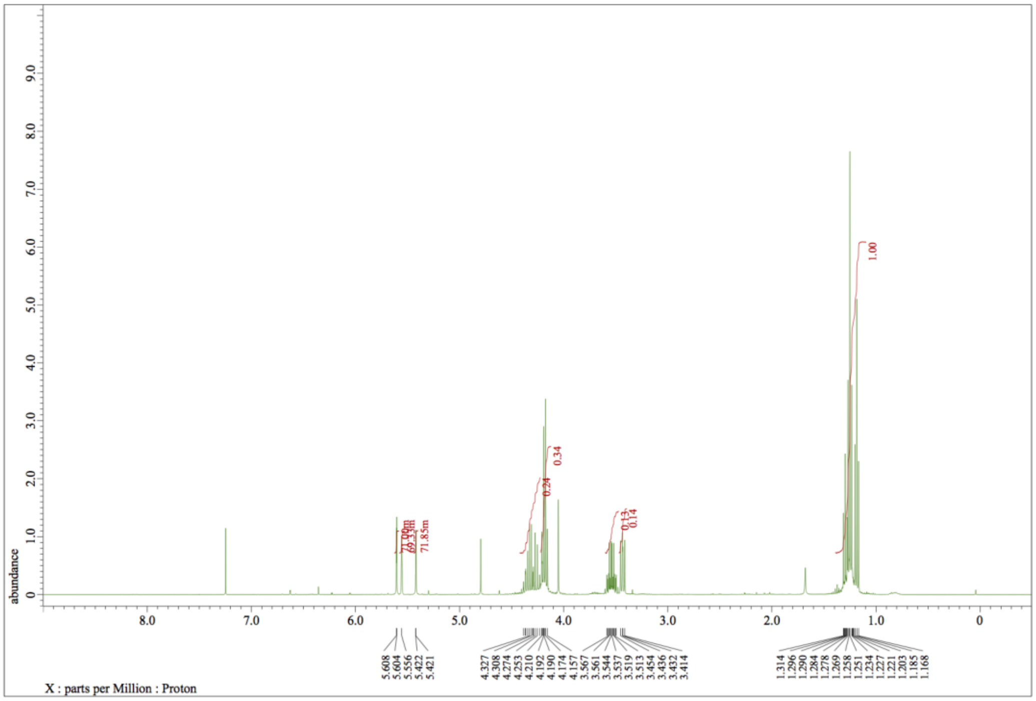
abundance
X : parts per Million : Proton
1.00
0.34
0.24
0.13
0.14
5.608
5.604
5.556
5.422
5.421
4.327
4.308
4.274
4.253
4.210
4.192
4.190
4.174
4.157
3.567
3.561
3.544
3.537
3.519
3.513
3.454
3.436
3.432
3.414
1.314
1.296
1.290
1.284
1.278
1.269
1.258
1.251
1.234
1.227
1.221
1.203
1.185
1.168

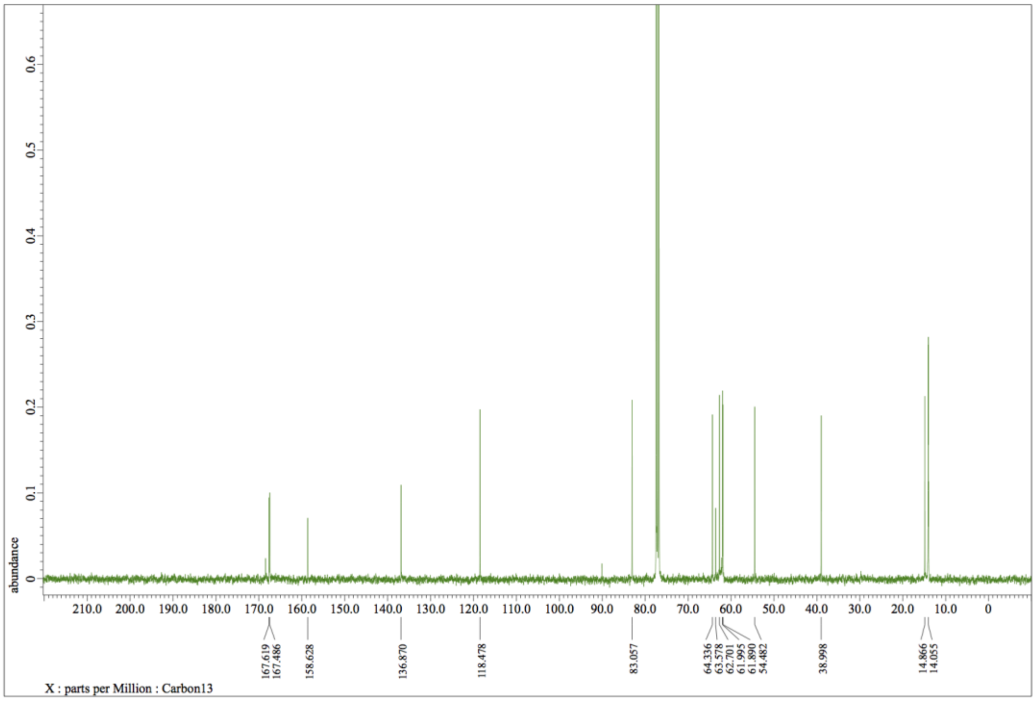
abundance
X : parts per Million : Carbon13
167.619
167.486
158.628
136.870
118.478
83.057
64.336
63.578
62.701
61.995
61.890
54.482
38.998
14.866
14.055

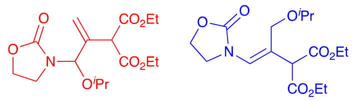

40a (1:3) **40b**

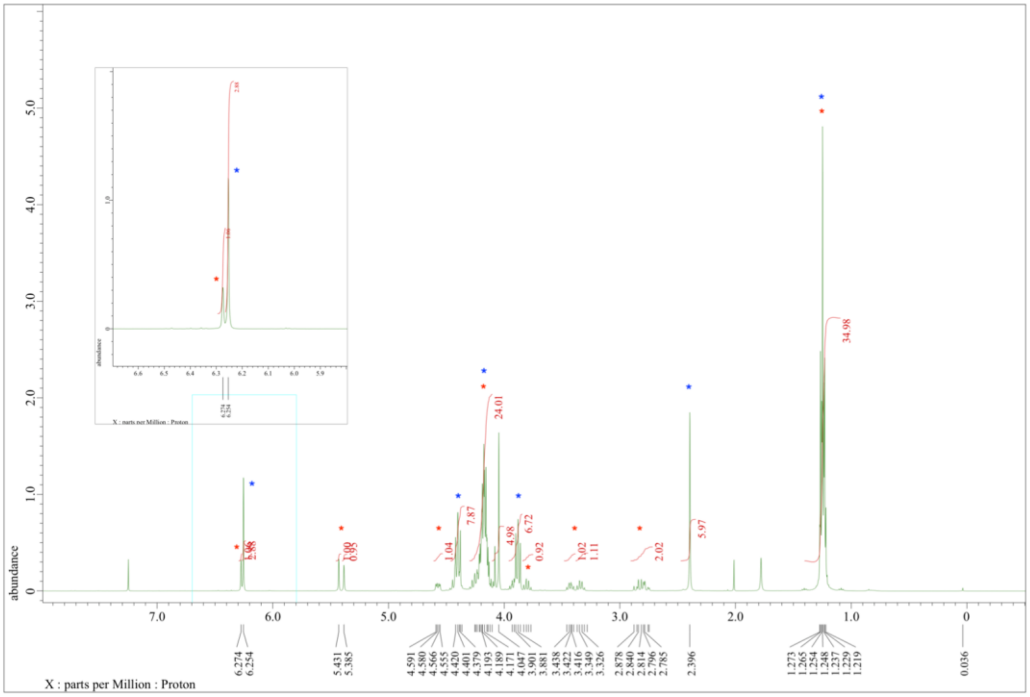

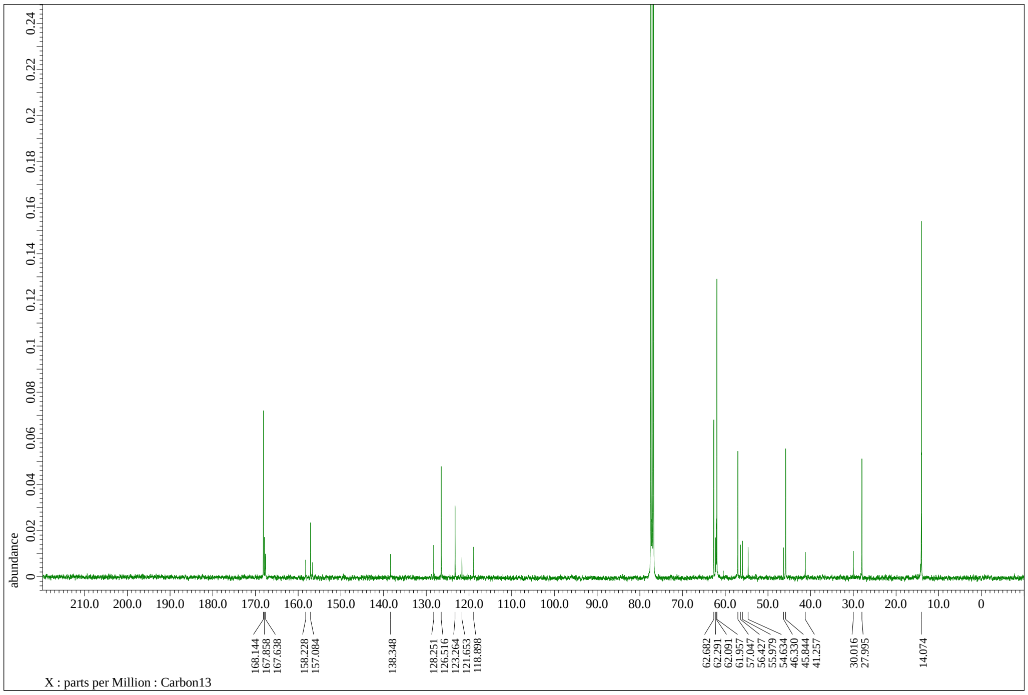

Supplement: File 1 — Experimental details, analytical (1H NMR, 13C NMR) and ESIMS data. [file Beilstein_J_Org_Chem-16-1983-s001.pdf]
